# Successful generation of epigenetic disease model mice by targeted demethylation of the epigenome

**DOI:** 10.1186/s13059-020-01991-8

**Published:** 2020-04-01

**Authors:** Takuro Horii, Sumiyo Morita, Shinjiro Hino, Mika Kimura, Yuko Hino, Hiroshi Kogo, Mitsuyoshi Nakao, Izuho Hatada

**Affiliations:** 1 Laboratory of Genome Science, Biosignal Genome Resource Center, Institute for Molecular and Cellular Regulation, Gunma University, 3-39-15 Showa-machi, Maebashi, Gunma 371-8512 Japan; 2 Department of Medical Cell Biology, Institute of Molecular Embryology and Genetics, Kumamoto University, 2-2-1 Honjo, Chuo-ku, Kumamoto, 860-0811 Japan; 3 Department of Anatomy and Cell Biology, Graduate School of Medicine, Gunma University, 3-39-22 Showa-machi, Maebashi, Gunma 371-8511 Japan

**Keywords:** CRISPR/Cas9, dCas9, Epigenome editing, Silver-Russell syndrome, Demethylation

## Abstract

**Background:**

Epigenetic modifications, including DNA methylation, play an important role in gene silencing and genome stability. Consequently, epigenetic dysregulation can cause several diseases, such as cancer, obesity, diabetes, autism, and imprinting disorders.

**Results:**

We validate three methods for the generation of epigenome-edited mice using the dCas9-SunTag and single-chain variable fragment-TET1 catalytic domain. We generate model mice for Silver-Russell syndrome (SRS), an imprinting disorder, by target-specific DNA demethylation in the *H19* differentially methylated region. Like SRS patients, these mice show *H19* upregulation and *Igf2* downregulation, leading to severe intrauterine and postnatal growth retardation.

**Conclusion:**

This is the first report of an imprinting disease model animal generated by targeted demethylation of specific loci of the epigenome in fertilized eggs. Epigenome-edited animals are also useful for exploring the causative epimutations in epigenetic diseases.

## Background

DNA methylation is a key epigenetic modification that plays an important role in gene silencing and genome stability [1–3]. Regions of hypermethylated DNA are usually associated with silenced and inactive chromatin, whereas regions of hypomethylated DNA are usually associated with gene expression and open chromatin. Compared with other epigenetic modifications, DNA methylation is thought to be relatively stable; however, it is sometimes affected by environmental change and aging, leading to epigenetic diseases such as cancer, obesity, diabetes, autism, and imprinting disorders [4–9]. Advances in DNA sequencing technologies have enabled genome-wide analysis of epigenetic information and supplied an enormous number of candidate disease-causing epigenetic changes [10–12]. However, before the development of epigenome editing, tools for directly demonstrating which epigenetic changes cause disease were not available. Epigenome editing at specific sites is achieved using engineered molecules targeted to those sites. Fusion proteins consisting of eukaryotic DNA methyltransferases or hydroxymethylation enzymes and DNA-binding proteins, such as zinc finger proteins [13], transcription activator-like effectors [14, 15], and catalytically inactive Cas9 (dCas9), based on the clustered regularly interspaced short palindromic repeat (CRISPR)/CRISPR-associated protein 9 (Cas9) system [16–18], have been used to produce targeted DNA modifications in vitro. This technology allows direct demonstration at the cellular level of the role of a candidate epigenetic gene in disease. Targeted DNA methylation has been achieved in mice by zygote microinjection using MQ1 DNA methyltransferase [19, 20] or DNA methyltransferase 3a (Dnmt3a) [21]. On the other hand, targeted DNA demethylation of hypermethylated regions is important for the reactivation of silenced genes; however, there have been few reports of successful targeted DNA demethylation of endogenous genes in fertilized eggs and generation of epigenome-edited animals [21].

**Fig. 1:**
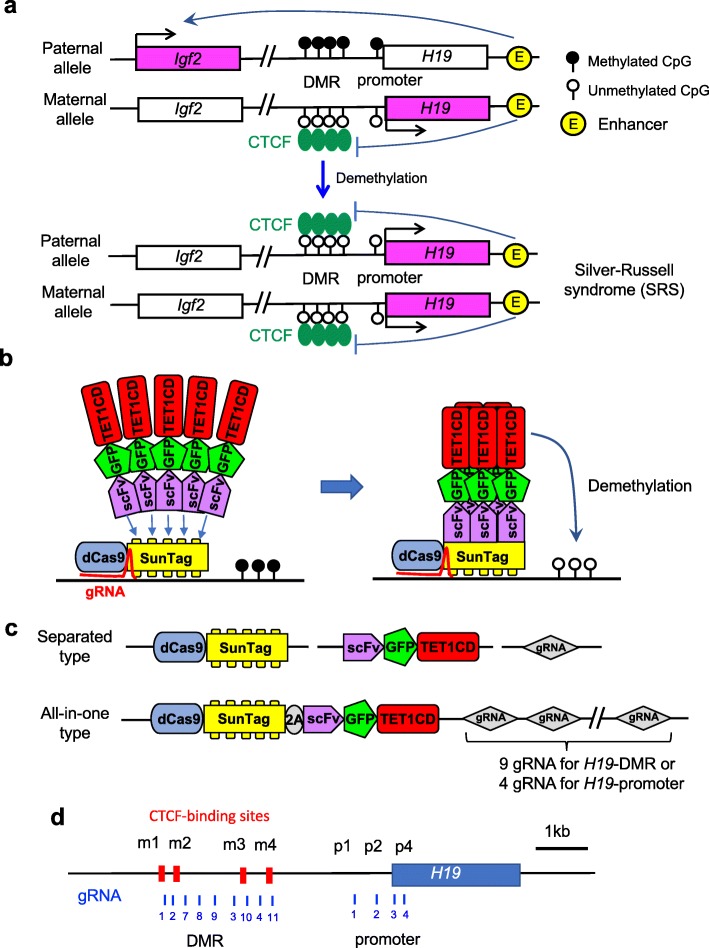
Schematics for targeted DNA demethylation of H19-DMR to generate a mouse model of human Silver-Russell syndrome (SRS). **a** In the normal situation in mouse and human, *Igf2* is expressed from the paternal allele, and *H19* is expressed from the maternal allele. In patients with SRS, DNA demethylation in *H19*-DMR results in biallelic expression of *H19* and repression of Igf2. **b** A scheme for CRISPR/Cas9- and SunTag-based induction of demethylation. dCas9 fused to a SunTag (multiple GCN4) can recruit multiple copies of antibody (scFv)-fused TET1CD. Thus, multiple copies of TET1CD hydroxylates specific loci and activates site-specific demethylation in the target. **c** Structure of vector components used in the experiments. The separated type was used for transient expression in ESCs. The all-in-one type was used for stable expression in embryos. **d** The mouse *H19* locus is shown with CTCF-binding sites indicated by red boxes. These CTCF-binding sites have methylation-sensitive CpG sites. Location of the targets for *H19*-DMR and *H19*-promoter used for gRNAs are indicated by blue bars. Scale indicates distance in kb

The imprinting disorder Silver-Russell syndrome (SRS) is a clinically and genetically heterogeneous condition characterized by severe intrauterine and postnatal growth retardation caused by reduction of insulin-like growth factor 2 (*Igf2*) gene expression [22–24]. Infants with this condition have low birth weight and often fail to grow and gain weight at the expected rate. Approximately 35–50% of patients with SRS show DNA hypomethylation of imprinting control center 1 between the *H19* and *Igf2* genes (*H19* differentially methylated region; *H19*-DMR) in the paternal allele [25]. *Igf2* and *H19* are reciprocally imprinted genes and are regulated by DNA methylation of the *H19*-DMR [26–28]. *Igf2* is expressed only from the paternal allele, and *H19* only from the maternal allele (Fig. 1a *upper*). The *H19*-DMR contains four highly conserved CG-rich repetitive sites (m1-m4) for binding CTCF, which has methylation-sensitive enhancer-blocking activity [29, 30]. In the paternal allele, methylation of CpGs within the CTCF-binding sites eliminates CTCF binding and results in loss of enhancer-blocking activity, thereby allowing *Igf2* expression. By contrast, hypomethylation of paternal *H19*-DMR in SRS allows CTCF binding, which leads to biallelic expression of *H19*, downregulation of *Igf2*, and hence growth retardation (Fig. 1a *lower*).

To generate model mice for SRS, we used an efficient epigenome editing system using dCas9 and SunTag [31] (Fig. 1b). dCas9 is a nuclease-deactivated variant of Cas9, which is used for site-specific targeting in the CRISPR/Cas9 system [32], and SunTag is a protein scaffold containing peptide epitopes able to recruit effector domains via specific single-chain variable fragment (scFv) antibodies [33]. The SunTag-carrying dCas9 recruits the scFv-green fluorescent protein (GFP)-TET1CD fusion protein, containing the catalytic domain (CD) of the ten-eleven translocation (TET) 1 hydroxylase, to the target locus, leading to targeted DNA demethylation [31]. The epigenome editing vectors and gRNAs used in this study are shown (Fig. 1c, d). Based on this system, we validated three methods using mouse embryonic stem cells (ESCs) or fertilized eggs for the generation of SRS model mice with targeted demethylation in *H19*-DMR. All three methods achieved target demethylation of *H19*-DMR and repression of *Igf2* genes.

## Results

### Method 1: Epigenome-edited mice derived from ESCs

**Fig. 2:**
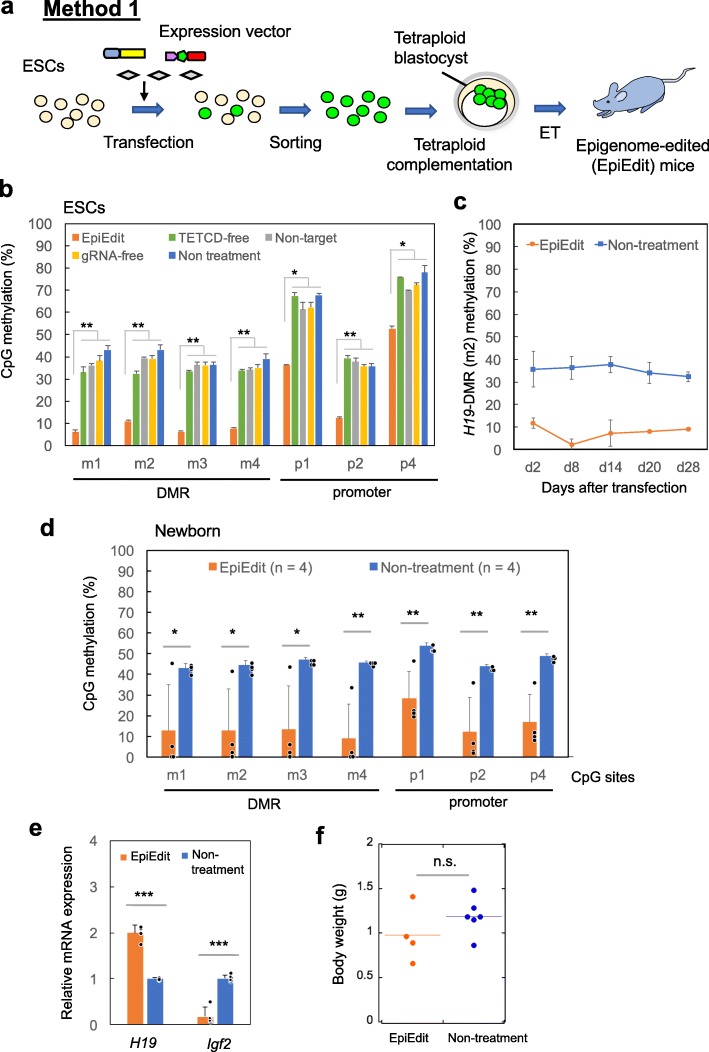
Generation of epigenome-edited (EpiEdit) mice via the tetraploid complementation method. **a** Schematic for generation of epigenome-edited ESC-derived mice. **b** Percentages of DNA methylation by COBRA in *H19*-DMR (m1–m4) and promoter (p1-p4) in undifferentiated ESCs (B6/J) at day 5 after transfection. Error bars, mean ± s.d. (biological triplicate). **c** Percentages of DNA methylation by COBRA in *H19*-DMR (m2) in undifferentiated ESCs (B6x129) during in vitro culture. d, days of culture after transfection. Error bars, mean ± s.d. (experimental triplicate). **d** Epigenome-edited newborn mice showed significant DNA demethylation at *H1*9-DMR (m1–m4) and promoter (p1-p4) by COBRA. **e** Expression analysis by qPCR. Epigenome-edited ESC-derived newborn mice showed upregulation of *H19* and downregulation of *Igf2* expression compared to control mice. **f** Epigenome-edited ESC-derived newborn mice did not show growth retardation. *n* indicates the number of mice used for the analysis. Error bars, mean ± s.d. * *P* < 0.05, **P < 0.01, ***P < 0.001, n.s., not significant (two-tailed Student’s *t* test)

First, we attempted to generate epigenome-edited mice using the tetraploid complementation method that enables generation of completely ESC-derived mice [34] (Fig. 2a). ESCs were transiently transfected with the epigenome editing vector targeting *H19*-DMR. After 2 days, GFP-expressing cells were sorted by fluorescent-activated cell sorting (FACS) to isolate vector-expressing cells. The epigenome-edited ESCs showed lower methylation levels in *H19*-DMR and promoter than in other control ESCs (Fig. 2b), and this demethylation was maintained for at least 4 weeks in culture (Fig. 2c). Epigenome-edited ESCs or non-treated control ESCs were introduced into the blastocoel cavity of tetraploid blastocysts, these embryos were transferred into uterine horns of pseudopregnant females, and then newborn mice were recovered. We obtained four mice derived from epigenome-edited ESCs and 13 mice derived from control ESCs (Additional file 1: Table S1). Average DNA methylation levels on the entire pup at *H19*-DMR (m1–m4 sites) were 9–13% in epigenome-edited mice and 43–47% in control mice (Fig. 2d). Of note, three out of four epigenome-edited mice showed almost complete demethylation of *H19*-DMR. Although ESCs were not transfected with the epigenome editing vector targeting *H19* promoter, epigenome-edited mice showed significant demethylation in the promoter region. In addition, epigenome-edited mice showed significant upregulation of *H19* and downregulation of *Igf2* expression due to *H19*-DMR demethylation (Fig. 2e). SRS patients usually have lower birth weights than healthy individuals due to the low expression of *Igf2*; however, there were no significant differences in body weight between epigenome-edited and control mice (Fig. 2f). This may be attributed to the lower litter size in epigenome-edited mice than that of control mice (1.3 vs 4.3). In general, fetal growth is inversely affected by the number of littermates [35]. Therefore, the lower number of littermates may have compensated for the reduction in fetal growth resulting from demethylation of *H19*-DMR. Another possibility is that intrauterine growth retardation in SRS patients may be driven by a placental phenotype (due to imprinted methylation errors) impacting the fetus; therefore, ESC-derived mice complemented by tetraploid embryos would not have this contribution to the phenotype.

### Method 2: Epigenome-edited mice by transient expression in fertilized eggs

**Fig. 3:**
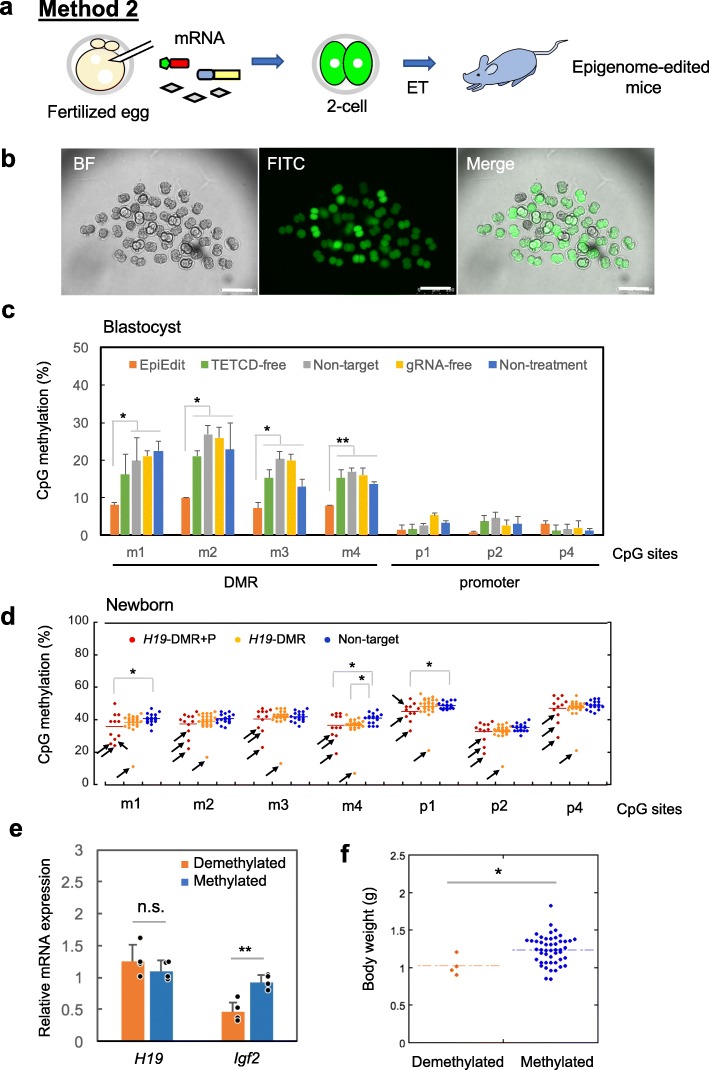
Generation of epigenome-edited mice by transient expression of epigenome editing factors in preimplantation embryos. **a** Schematic for generation of epigenome-edited mice. In vitro transcribed mRNA was introduced into zygotes. **b** Epigenome editing factors (GFP) were expressed in almost all embryos 1 day after introduction. Scale bars, 200 μm. **c** Percentages of DNA methylation by COBRA in *H19*-DMR (m1-m4) and promoter (p1-p4) in epigenome-edited (epiedit) blastocyst (gRNAs for both *H19*-DMR and *H19*-promoter) and various control blastocysts. Non-target control was generated by introduction of gRNA for *RASSF1A* (human specific sequence). Blastocyst sample introduced all components shows significant demethylation in *H19*-DMR compared to other control sample. **d** Epigenome-edited newborn mice were generated by introduction of gRNAs for both *H19*-DMR and *H19*-promoter (*H19*-DMR + P, red dot) or gRNAs for only *H19*-DMR (yellow dot). Most newborn mice did not show a change in methylation, whereas four mice (*arrows*) showed apparent demethylation compared with control mice. **e** Expression analysis by qPCR. The four demethylated newborn mice showed downregulation of *Igf2* expression compared with methylated control mice. **f** The four demethylated newborn mice showed significantly lower body weight than methylated control mice. Error bars, mean ± s.d. *P < 0.05, **P < 0.01, n.s., not significant (two-tailed Student’s *t* test)

Generation of epigenome-edited mice using epigenome-edited ESCs is a powerful tool because almost all animals obtained showed demethylation in *H19*-DMR. However, tetraploid complementation methods can be applied only to rodents because totipotent ESC lines have not been established in other animal species. To generalize our epigenome editing system to other animal species, we next examined the introduction of mRNA for epigenome editing in preimplantation embryos (Fig. 3a). In this experiment, epigenome editing factors including gRNA for only *H19*-DMR or both *H19*-DMR and *H19*-promoter regions were introduced into fertilized eggs. According to the GFP intensity, epigenome editing factors were strongly expressed in almost all embryos at the two-cell stage (Fig. 3b). The methylation levels of *H19*-DMR (m1-m4) were lower in epigenome-edited blastocysts generated by introduction of gRNAs for both *H19*-DMR and *H19*-promoter regions than in other control blastocysts (Fig. 3c). In contrast, *H19*-promoter region was hypomethylated in all samples at blastocyst stage. In general, DNA methylation spreads in *cis* from *H19*-DMR to the promoter during postimplantation development [28]. DNA demethylation levels in *H19*-DMR were correlated with the expression levels (GFP intensities) of epigenome editing factors (Additional file 2: Fig. S1). Following embryo transfer, we obtained only four mice apparently showing demethylation in *H19*-DMR (Fig. 3d and Additional file 1: Table S2). In contrast with epigenome-edited ESC-derived mice, almost all pups generated by the transient expression method showed normal methylation levels. In this experiment, we introduced gRNAs for both *H19*-DMR and *H19*-promoter simultaneously to improve the editing efficiency; nevertheless, the percentage of methylation in *H19*-DMR was still high. Although there were no significant differences in body weight between any group (data not shown), four demethylated mice in *H19*-DMR showed downregulation of *Igf2* expression due to *H19*-DMR demethylation (Fig. 3e). In contrast, *H19* expression level is comparable between demethylated mice and the control mice. Although the reason for this is not clear, only one mouse showed robust demethylation of *H19*-DMR in method 2 (Fig. 3d) while more than one mouse showed robust demethylation in method 1 (Fig. 2d). These four demethylated mice also showed reduction of body weight at birth (Fig. 3f).

### Method 3: Epigenome-edited mice by stable expression in fertilized eggs

**Fig. 4:**
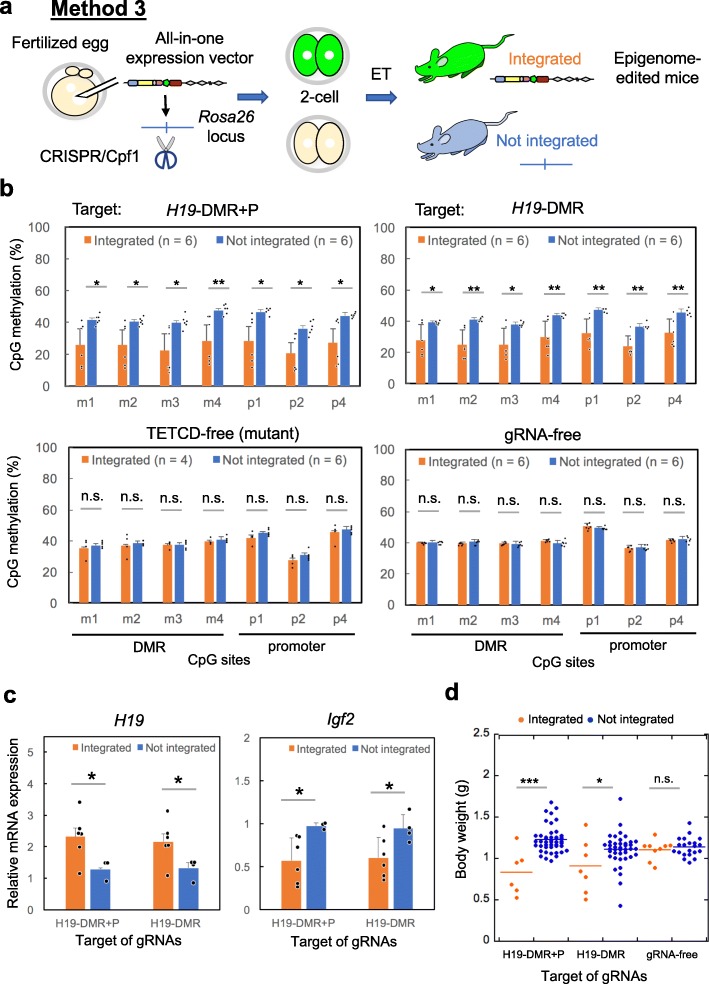
Generation of epigenome-edited mice by stable expression of epigenome editing factors in mice. **a** Schematics for generation of epigenome-edited mice. Linearized all-in-one vector (Fig. 1c) was introduced into the Rosa26 locus, which was cut by the CRISPR/Cpf1 system. **b** Epigenome-edited newborn mice were generated by introduction of a vector expressing gRNAs for both *H19*-DMR and *H19*-promoter (H19-DMR + P) or gRNAs for only *H19*-DMR. Control mice were generated by introduction of vector with TETCD mutant (TETCD-free) or vector without gRNAs (gRNA-free). Significant demethylation in *H19*-DMR and promoter regions was observed in the *H19*-DMR+P and *H19*-DMR integrated newborn mice. Interestingly, the mice in which only gRNAs for *H19*-DMR were introduced also showed demethylation in the *H19*-promoter region. **c** Expression analysis by qPCR. Vector-integrated newborn mice showed upregulation of *H19* and downregulation of *Igf2* expression. **d** Reduction of body weight was observed in newborn mice in which the vector with *H19* gRNAs was integrated. *n* indicates the number of mice used for the analysis. Error bars, mean ± s.d. * *P* < 0.05, **P < 0.01, ***P < 0.001, n.s., not significant (two-tailed Student’s *t* test)

Transient expression of epigenome editing factors may be insufficient to obtain epigenome-edited mice with efficient demethylation. Indeed, the transient signal of epigenome editing factors had mostly disappeared by the blastocyst stage (Additional file 2: Fig. S2). To maintain demethylated status during development, we next examined stable expression of epigenome editing factors using a transgenic strategy. In brief, a linearized all-in-one vector for epigenome editing was introduced into the *Rosa26* locus in fertilized eggs, and phenotypes were compared between mice with or without vector integration (Fig. 4a and Additional file 1: Table S3). According to the GFP intensity, epigenome editing factors were indeed expressed in preimplantation embryos and newborn mice (Additional file 2: Fig. S3). In total, 12–36% of newborn mice exhibited vector integration, and 50–67% of these mice showed significant demethylation at seven CpG sites in *H19*-DMR and *H19*-promoter regions examined by COBRA (Fig. 4b). By contrast, mice with integration of a control vector, including catalytically dead TET1CD or the control gRNA-free vector, did not show any demethylation at these sites (Fig. 4b). Vector-integrated mice showed upregulation of *H19* and downregulation of *Igf2* genes (Fig. 4c). In addition, vector-integrated mice had significantly lower body weights than mice without vector integration (Fig. 4d). By contrast, mice integrated with the control vector (gRNA-free vector) did not show any change in gene expression (Additional file 2: Fig. S4a) or body weight (Fig. 4d). We observed strong correlations between *H19*-DMR methylation, *Igf2* expression, and body weight (Additional file 2: Fig. S4b–d). In some cases, no difference in DNA methylation at *H19*-DMR could be detected between vector-integrated mice and control mice, with these vector-integrated mice having no expression of dCas9 (Additional file 2: Fig. S4e, arrows). Unexpectedly, PCR analysis clarified that the vector was not integrated into the *Rosa26* locus but other loci in all vector integrated mice (Additional file 2: Fig. S5), indicating variation of dCas9 expression was caused by vector-integrated loci. To determine whether CTCF binding was affected in epigenome-edited mice, cells derived from whole tissues of newborn mice were analyzed by chromatin immunoprecipitation (ChIP)-qPCR to detect CTCF binding. The experimental results consistently demonstrated that CTCF binding to the targeted site was significantly increased at the m2 site of *H19*-DMR, the site at which we observed decreased methylation (Additional file 2: Fig. S6). CTCF binding did not differ between the upstream region (*H19*-DMR-5′) and the downstream region (*H19* promoter) where CTCF did not bind. Furthermore, CTCF binding was maintained at the CTCF binding site at the *Hox A* locus where targeted demethylation was not induced.

**Fig. 5:**
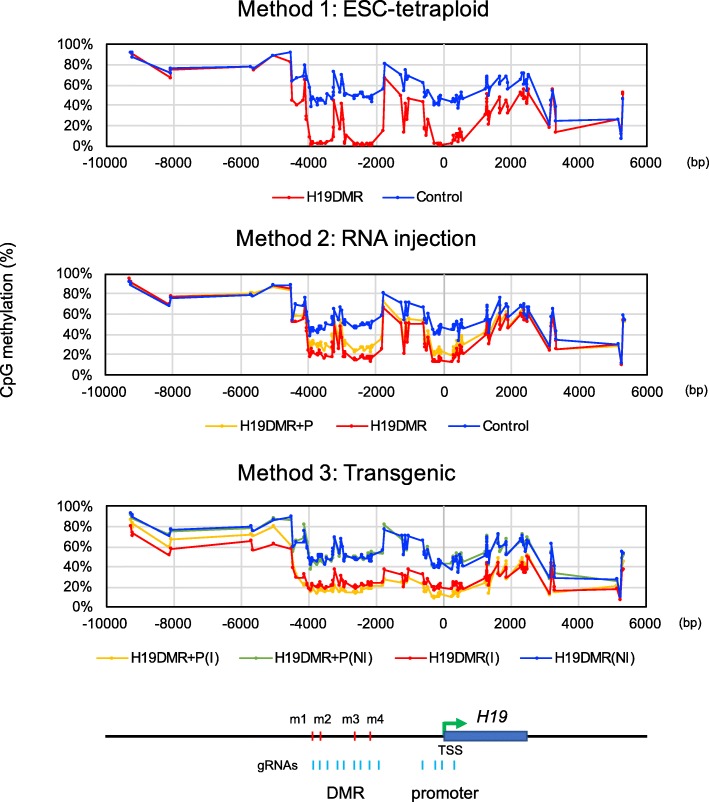
Comprehensive DNA methylation analysis around *H19*-DMR. Bisulfite amplicon sequencing was performed for DNA methylation analysis of representative newborn mice generated by three methods. PCR products were amplified using 24 primer pairs covering 145 CpG sites across 15-kb regions around *H19*-DMR. I, vector integrated; NI, not integrated; TSS, transcription start site

To compare comprehensive DNA methylation status among the three methods, bisulfite amplicon sequencing analysis was performed for 145 CpG sites across 15-kb regions around *H19*-DMR (Fig. 5). *H19*-DMR, promoter, and gene body regions were hypomethylated in all epigenome-edited newborn mice as expected. Among these samples, the DNA methylation pattern was similar and did not depend on gRNA (*H19*-DMR or *H19*-DMR + promoter) or method used. We also assessed the off-target effects of all three methods for all potential off-target regions (6 regions) in the genome with less than 4 mismatches for used 13 gRNAs. In addition, we examined each 2 potential off-target regions with 4, 5, and 6 mismatches, respectively. As a result, we found demethylation in the 2 potential off-target regions for gRNA of H19DMR_10 (2 mismatch) and H19DMR_11 (2 mismatch) in the method 3 sample. This indicates that stable expression of epigenome editing factors could increase the risk of off-target epigenome editing (Additional file 2: Fig. S7).

**Fig. 6:**
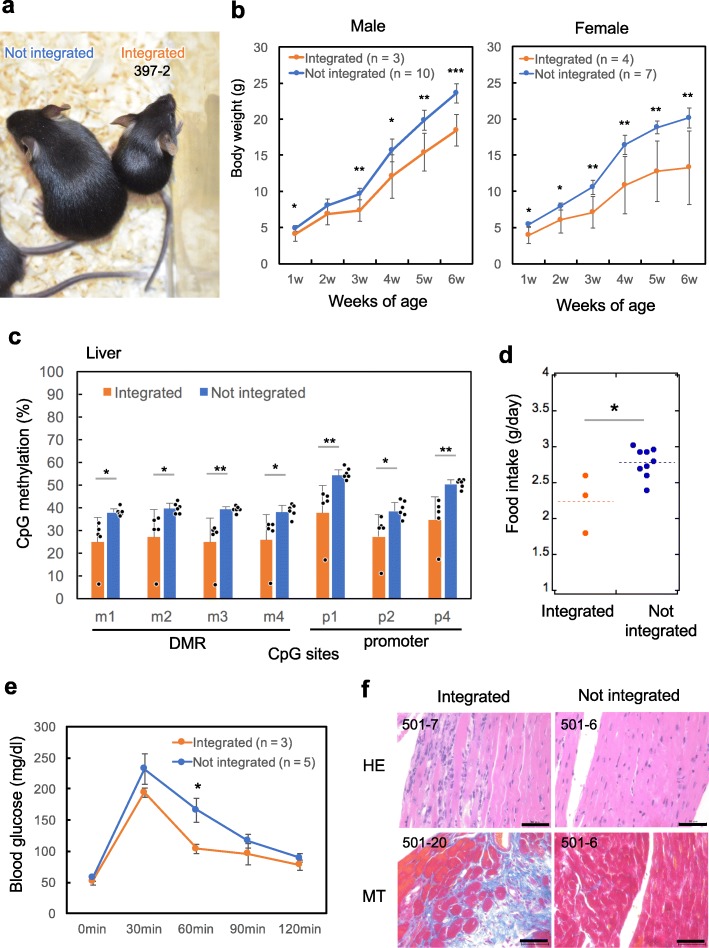
Postnatal development and phenotype of SRS modeling mice. **a** Appearance of vector-integrated and control female mice at 4 weeks of age. **b** Growth retardation was observed in vector-integrated mice. *n* indicates the number of mice used for the analysis. Error bars, mean ± s.d. *P < 0.05, **P < 0.01, ***P < 0.001 (two-tailed Student’s *t* test). **c***H19*-DMR methylation status analyzed by COBRA in liver at 8 weeks of age. Vector-integrated mice frequently showed demethylation. Error bars, mean ± s.d. *P < 0.05, **P < 0.01 (two-tailed Student’s *t* test). **d** Food intake (g/day) was examined in vector integrated and not integrated female mice (3 weeks of age). *P < 0.05 (two-tailed Student’s *t* test). **e** Glucose tolerance tests were performed after an overnight fast in vector integrated and not integrated male mice (7 weeks of age) fed normal chow. Blood glucose concentration appears to be lower in epigenome-edited mice. *P < 0.05 (one-tailed Student’s *t* test). Error bars, mean ± s.d. **f** Vector-integrated mice showed degeneration (2/3) and fibrosis (3/3) in cardiac muscle fiber whereas vector non-integrated mice did not show these phenotypes (0/3). HE, hematoxylin and eosin stain; MT, Masson’s trichrome stain. Scale bars: 50 μm

**Fig. 7:**
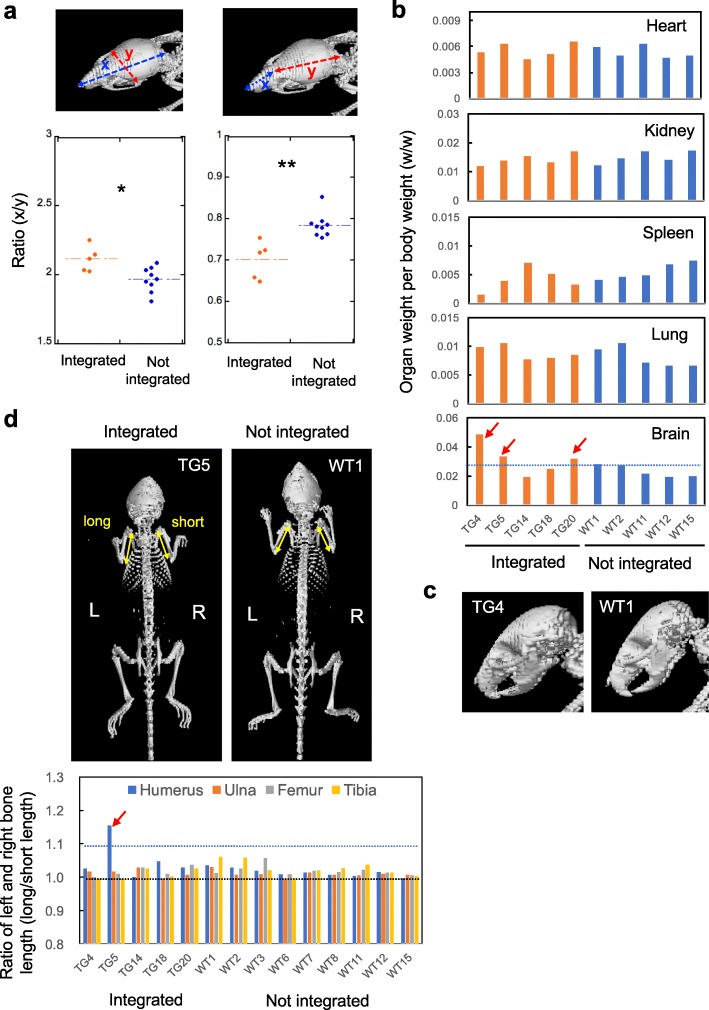
Craniofacial abnormality, relative macrocephaly, and body asymmetry observed in SRS model mice. **a** Craniofacial feature was examined by 3D image reconstructed from CT scan of a mouse. Linear measurements obtained from each pair of landmarks (nasal bone, edge of occipital bone, glabella, and lateral angle of eye) were shown by arrows. The ratio of two parameters (*x*/*y*) was compared between vector integrated and not integrated mice. * *P* < 0.05, ** *P* < 0.01 (two-tailed Student’s *t* test). **b** Organ weight per body weight (w/w) of vector integrated and not integrated mice. Increase of relative brain weight (w/w) associated with macrocephaly was observed in vector-integrated mice (arrows). **c** 3D images of a mouse with relative macrocephary (TG4) and a control mouse (WT1) are shown. **d** 3D image reconstructed from CT scan of a mouse which showed body asymmetry in the humerus bone (TG5) and a control mouse (WT1). The ratio of right and left limb bone lengths was calculated from 3D image of a mouse. The bar graphs show the ratio of the long length to the short length. Body asymmetry was observed in the humerus bone of epigenome-edited mice (TG5, *arrow*)

We also examined postnatal growth of epigenome-edited mice in which gRNA for *H19*-DMR vector was introduced. Like SRS patients, weight gain after birth was severely restricted in the epigenome-edited mice (Fig. 6a,b). *H19*-DMR hypomethylation was observed in the liver of adult mice (Fig. 6c). In addition, epigenome-edited mice exhibited low food intake (g/day) at 3 weeks of age, and both hypoglycemia and muscle fiber degeneration/fibrosis in cardiac muscle at 7–8 weeks of age (Fig. 6d–f). Feeding difficulties, hypoglycemia, and heart disease are observed in 84%, 24%, and 9% of SRS cases, respectively [36]. Although fiber degeneration and fibrosis in cardiac muscle have not been clinically reported as far as we know [36–38], it is interesting to note that *H19* is involved in cardiac fibrosis [39], indicating that this is an overlooked phenotype of SRS. Detailed analysis of bones was performed using micro-computed tomography (micro-CT). Epigenome-edited mice showed craniofacial features like elongated forehead which could be associated with triangular face and prominent forehead observed in SRS patients (Fig. 7a). Relative brain weight per body weight (w/w) indicated that macrocephaly occurred in some of SRS model mice (Fig. 7b,c). In addition, one of five epigenome-edited mice exhibited body asymmetry in humerus bone (Fig. 7d); however, the incidence rate in SRS model mice was much lower than that in SRS patients (20% vs 68%), and the relationship with *H19*-DMR demethylation is unclear at present. On the other hand, epigenome-edited mice did not show apparent clinodactyly (Additional file 2: Fig. S8a) and showed normal spermatogenesis (Additional file 2: Fig. S8b). To demonstrate germline transmission of the *H19*-DMR demethylation in mice which stably express the epigenome editing factors, DNA methylation status in *H19-*DMR and promoter was analyzed using F1 offspring (e18.5) derived from a vector-integrated female founder mouse. The results of COBRA showed apparent demethylation in vector-integrated F1 mice (Additional file 2: Fig. S9).

### Generation of an SRS model from human cells

**Fig. 8:**
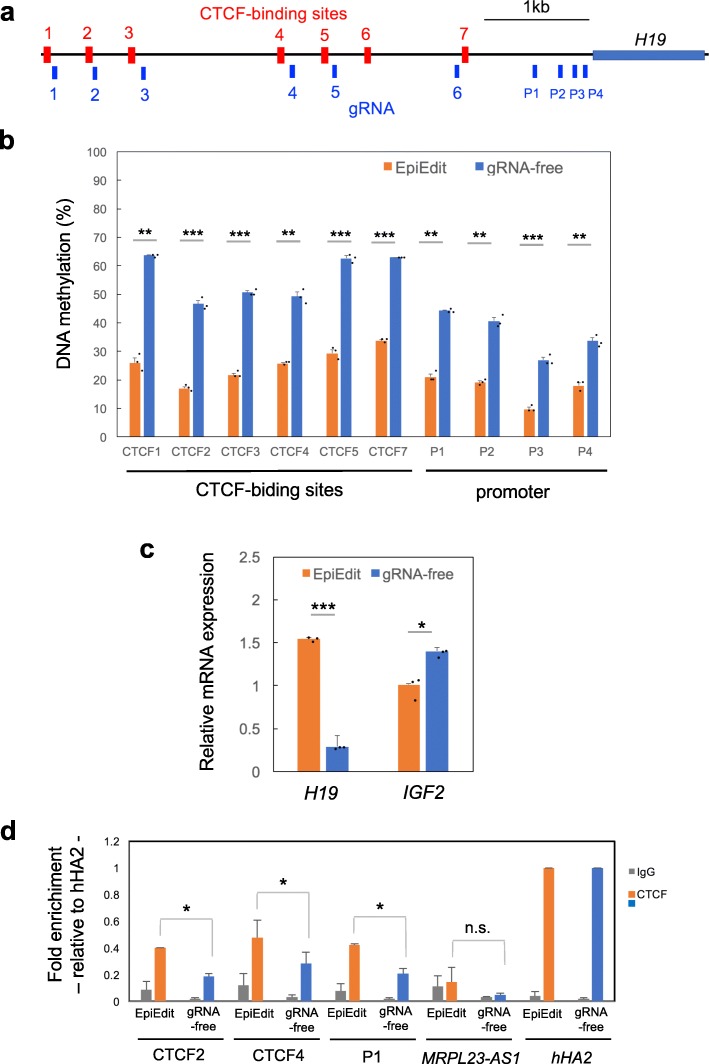
Generation of SRS model from human cell line HEK293. **a** The human *H19* locus is shown with CTCF-binding sites indicated by red boxes. These CTCF-binding sites have methylation-sensitive CpG sites. Location of the targets for *H19*-DMR and *H19*-promoter used for gRNAs are indicated by blue bars. Scale indicates distance in kb. **b** Percentages of DNA methylation by COBRA at day 5 after transfection. Error bars, mean ± s.d. (biological triplicate). **c** Expression analysis by qPCR. SRS model cell lines showed upregulation of *H19* and downregulation of *IGF2* expression compared to control cells. Error bars, mean ± s.d. (biological triplicate). *P < 0.05, **P < 0.01, ***P < 0.001 (two-tailed Student’s *t* test). **d** Anti-CTCF ChIP was performed using cells at day 9 after transfection followed by qPCR analysis. Targeting demethylation in *H19*-DMR increases CTCF-binding to the CTCF2, CTCF4, and P1 sites. *MRPL23-AS1* was selected as negative control, and the CTCF binding site within *Hox A* gene cluster (*hHA2*) was selected as positive control. Data are normalized by *hHA2*. bars, mean ± s.d. (biological triplicate). *P < 0.05, n.s., not significant (one-way ANOVA)

Finally, we applied this system to generate SRS model cell lines using HEK293 human embryonic kidney cells. For this purpose, HEK293 cells were transfected with the epigenome editing vector containing six gRNAs for CTCF-binding sites [40] and four gRNAs for the promoter region of *H19* (Fig. 8a). Five days after transfection, cells were harvested and the DNA methylation status were analyzed. As expected, vector-transfected cells showed significant CpG demethylation at the six CTCF-binding sites (except for CTCF6) and the promoter region of *H19* (Fig. 8b). We did not examine the methylation level at the CTCF6 site because this site is normally hypomethylated [40]. Vector-transfected cells exhibited upregulation of *H19* and downregulation of *IGF2* transcripts (Fig. 8c). CTCF binding was significantly increased at the CTCF2 and CTCF4 sites of *H19*-DMR, the sites at which we observed decreased methylation (Fig. 8d). In contrast with the results of mouse ChIP-qPCR, CTCF binding was also observed at the *H19* promoter region P1, indicating that this is a specific characteristic of HEK293 cells. Hence, generation of a SRS model by targeted demethylation of *H19*-DMR was successfully achieved in both mice and a human cell line.

## Discussion

In this study, we produced epigenome-edited animal models using mouse ESCs and fertilized eggs. Using all three methods, we achieved targeted demethylation of *H19*-DMR with transcriptional changes at the *Igf2* gene in newborn mice. To the best of our knowledge, this is the first report of epigenetic disease model mice in which targeted DNA demethylation was induced in preimplantation embryos. A previous report showed that targeted demethylation of neural precursor cells derived from induced pluripotent stem cells (iPSCs) with chromosomal integration of the dCas9-TET system were transplanted to the postnatal day 1 mouse brain, and edited methylation status was maintained 1 month post-engraftment in vivo [41]. Xu et al. demonstrated successful targeted demethylation of the *Rasal1* and *Klotho* genes by in vivo lentiviral delivery to decrease fibrosis in a mouse model [42]. However, these experiments were performed using adult mice, and it was unclear whether the demethylation-targeted epigenome of imprinted genes in fertilized eggs could be maintained after the preimplantation stage and persist in the animal.

In contrast, there are some studies reporting that targeted DNA methylation can be achieved by using fertilized eggs with introduction of transcription activator-like effectors or dCas9 directly fused to a *Sss* I or MQ1 methyltransferase [19, 20]. For example, when DNA methylation of *H19* CTCF-binding sites, the same locus as in the current study, was targeted using dCas9 directly fused to a MQ1 methyltransferase, methylation changes were detected at only half of the CTCF-binding sites (m3 and m4) [19]. Accordingly, no significant changes in weight were observed in the methylation-induced mice, whereas a previous study reported that full methylation of these four CTCF-binding sites led to gains in birth weight and adult body mass [43]. By contrast, in our study using DNA demethylation at the same locus, demethylation of all four CTCF-binding sites (m1-m4) led to aberrant *Igf2* and *H19* expression with resultant reductions in birth weight and adult body mass. Several possibilities can be advanced to explain these discrepancies between previous reports and our study. One possibility is that the induced epigenetic changes reported in previous studies were a consequence of changes in methylation, but those reported in our study were a consequence of changes in demethylation. Complete methylation of the four CTCF-binding sites is necessary for transcriptional repression of *H19* [19], but it is possible that partial demethylation is enough for its transcriptional activation. In other words, transcriptional activation by targeted demethylation might occur more easily than transcriptional repression by targeted methylation. Another possibility might be differences between the epigenome editing systems reported previously and the system used in our study. In one report, targeted methylation was achieved by fusing dCas9 directly to a MQ1 protein [19]; however, this only recruits one copy of MQ1 protein to the target site. By contrast, we used the SunTag system which recruits multiple copies of TET1 protein to the target site to achieve efficient and broad demethylation of *H19*-DMR.

In this study, we compared three methods for targeted demethylation of an endogenous gene in mice. Although all three methods can yield epigenome-edited animals, each method has advantages and disadvantages as follows. Method 1: Generation of epigenome-edited ESC-derived animals by tetraploid complementation can be applied only to rodents, but the extent of demethylation in almost all animals obtained was higher than with the other two methods. Highest frequency (75%) of newborns had *H19-*DMR demethylation and newborns not likely to be mosaic. ESC-derived animals cannot discern placental contributions to the disease phenotype. Epigenetic changes of genomic imprinting induced by epigenome editing would not be inherited by the next generation because these epigenetic marks are erased through the germ-lineage [44]. Method 2: Generation of epigenome-edited animals by transient mRNA expression of epigenome editing factors could be applied to most animal species. However, epigenome-edited animals were obtained with low frequency and with a low degree of demethylation. Embryos/fetuses/newborns may be mosaic due to targeting at or after the two-cell stage. Epigenetic changes of genomic imprinting induced by epigenome editing would not be inherited by the next generation. Method 3: Epigenome-edited animals by stable expression of epigenome editing factors could also be applied to most animal species. High frequency (50–67%) of vector-integrated newborns had *H19*-DMR demethylation and can discern placental contributions to the disease phenotype. Low integrated vector targeting *H19*-DMR (12–13%) in newborns and embryos/fetuses/newborns may be mosaic due to targeting at or after the two-cell stage. Epigenetic change would be inherited to the next generation because of vector integration. This makes it possible to establish permanent epigenetic disease mouse models.

Although all three methods can yield epigenome-edited animals, *H19*-DMR methylation levels were variable in each method. In the case of method 1 (epigenome-edited mice derived from ESCs), three mice showed dramatic changes in methylation and the fourth showed subtle changes. In general, ESCs are characterized by high cellular heterogeneity in epigenetic modifications including DNA methylation [45, 46]. ESCs after epigenome editing also seem not to be clonal but to be heterogeneous with respect to DNA methylation status; therefore, the methylation status of a given mouse could depend on which cell that contributed to the tissue under study. Similar results have been observed with other strategies. In the case of method 2 (epigenome-edited mice produced by transient mRNA expression), epigenome editing factors were strongly expressed in almost all embryos at the two-cell stage and hypomethylation of *H19*-DMR was observed at the blastocyst stage; nevertheless, epigenome-edited mice were obtained with low frequency and with a low degree of demethylation. One explanation for this is that these embryos exhibit epigenetic mosaicism, and cells that evade targeted demethylation in an embryo may gain a survival advantage during in utero development due to higher expression of *Igf2*, which is critical for cell growth. In the case of method 3 (epigenome-edited mice produced by the stable expression), the success of this approach depends on whether the epigenome editing vector is integrated or not. In fact, mice without vector integration rarely showed DNA methylation changes. GFP expression was equivalent in both blastomeres of two-cell embryos following RNA injection, while only one of the two blastomeres in several two-cell embryos was GFP-positive following vector integration (Additional file 2: Fig. S3a). This indicates that the vector could only be successfully integrated into one blastomere in these embryos. In this case, the embryo would exhibit epigenetic mosaicism. Even vector-integrated mice sometimes did not show DNA demethylation of *H19*-DMR. This could be caused by silencing of the inserted vector via DNA methylation [47] and/or chromatin modification [48]. Indeed, vector-integrated mice not showing *H19*-DMR demethylation had no expression of dCas9 (Additional file 2: Fig. S4e, arrows).

Our results indicate that the introduction of only gRNAs for *H19*-DMR was enough to reactivate *H19* and reduce *Igf2* expression (Fig. 4c), indicating that *H19*-DMR but not the *H19-*promoter is essential for regulation of *Igf2*/*H19* expression. In general, *H19*-DMR inherits paternal-specific methylation from sperm, which regulates the *H19* gene during preimplantation development. During early postimplantation development, DNA methylation and heterochromatin formation spreads in *cis* from *H19*-DMR to the *H19* promoter, thereby stably and permanently silencing the paternal *H19* allele [28, 49]. Our results are consistent with these previous reports.

## Conclusions

This study demonstrates directly that targeted demethylation of *H19*-DMR induces an SRS-like phenotype, including reduced body weight. Targeted demethylation technology could be applied to the modeling of other imprinting disorders, such as Angelman syndrome, which is occasionally caused by loss of maternal methylation at the *SNRPN* imprinting control region [50–52]. Epigenetic anomalies with DNA hypomethylation are expected to cause several diseases; therefore, the successful generation of mice with targeted DNA demethylation at certain loci will be useful for exploring the causative epimutations in epigenetic diseases. In addition, these mice could be used as disease models.

## Methods

### Animals

B6D2F1 mice were purchased from CLEA Japan (Kawasaki, Japan). C57BL/6J and ICR mice were purchased from Charles River Japan (Yokohama, Japan). 129X1/svJJmsSlc mice were purchased from Japan SLC (Shizuoka, Japan).

### Vector construction for targeted demethylation

We have previously reported the dCas9 expression vector fused with five copies of GCN4 (pCAG-dCas9-5xPlat2AflD; Addgene plasmid 82560) and the expression vector for anti-GCN4 peptide antibody (scFv)-superfolder green fluorescent protein (sfGFP)-TET1CD fusion protein (pCAG-scFvGCN4sfGFPTET1CD; Addgene plasmid 82561) [31]. The gRNA vectors for *H19*-DMR and *H19*-promoter were generated by inserting the target sequences into gRNA cloning vector (Addgene plasmid 41824). Cloning was performed by linearization of an *Afl*II site and Gibson assembly-mediated incorporation of the gRNA insert fragment. The gRNA target sequences are described (Additional file 1: Table S4 and S5). By contrast, two all-in-one vectors for *H19*-DMR (pPlatTET-gRNA2-H19DMRx9) and *H19*-promoter (pPlatTET-gRNA2-H19P) are based on pPlatTET-gRNA2 (Addgene plasmid 82,559). Detailed plasmid maps are shown (Additional file 2: Fig. S10–S12). Each all-in-one vector was linearized by *Apa*LI before use.

### In vitro transcription of RNA

pCAG-dCas9-5xPlat2AflD, pCAG-scFvGCN4sfGFPTET1CD, and gRNA vectors were amplified by Q5 Hot Start High-Fidelity DNA Polymerase (New England BioLabs) using primer sets for in vitro transcription (Additional file 1: Table S4). The amplified PCR product was gel purified and used as the template for in vitro transcription. MEGAshortscript T7 Kit (Life Technologies) was used for gRNA, and mMESSAGE mMACHINE T7 ULTRA kit (Life Technologies) was used for others. In vitro transcribed RNA was eluted into RNase-free water, and the quality was checked by gel electrophoresis.

### Epigenome editing of cultured cell lines

ESCs were established from mouse (C57BL/6 J or C57BL/6J x 129X1/svJJmsSlc) blastocysts. ESCs were cultured at 37 °C under 5% CO_2_ in Dulbecco’s modified Eagle’s medium (DMEM)-high glucose (D6429-500ML, Sigma) supplemented with 1% fetal bovine serum (FBS), 17.5% KSR (10828028, Gibco), 0.2% 2-mercaptoethanol (21985-023, Gibco), and 1 × 10^3^ units/mL ESGRO mouse LIF (ESG1107, Millipore). HEK293 cells (RIKEN BRC) were cultured in DMEM supplemented with 10% FBS. Cells were transfected with Lipofectamine 2000 (Invitrogen) according to the manufacturer’s protocols, harvested 48 h later, and sorted using FACSAriaII (BD Biosciences). The molar ratio of the dCas9-peptide array fusion vector, scFv-GFP-TET1CD vector, and gRNA vector in the transfection was 1:2:4, respectively. The ESCs were used for the tetraploid complementation experiment after 12 days in culture.

### Preparation of embryos

B6D2F1 female mice (8–10 weeks old) were induced to superovulate by injecting 7.5 units of pregnant mare’s serum (SEROTROPIN; ASKA Pharmaceutical, Tokyo, Japan) followed 48 h later with 7.5 units of human chorionic gonadotropin (hCG; GONATROPIN, ASKA Pharmaceutical). After administration of hCG, females were mated with B6D2F1 males. Zygotes were isolated from the oviduct 21 h later. After treatment in M2 medium (Sigma-Aldrich, St. Louis, MO, USA) supplemented with 0.1% hyaluronidase (Sigma-Aldrich) in a few minutes, fertilized eggs were transferred to drops of M16 medium (Sigma-Aldrich) supplemented with penicillin and streptomycin at 37 °C.

### Tetraploid complementation

The blastomeres of two-cell embryos were electrofused to produce tetraploid embryos 42 h post hCG treatment. Two-cell embryos were washed in 0.3 M Mannitol medium supplemented with 0.5 mM CaCl_2_ and 0.1 mM MgSO_4_, and transferred to an electrode chamber (LF501PT1–10; BEX, Tokyo, Japan) filled with 5 μl of Mannitol medium and connected with an CFB16-HB electroporator (BEX). Two-cell embryos were electrified with 10 V for 2 s to align the embryos parallel to the electrode and with 200 V for 40 μs to electrofuse the embryos. After electrofusion, embryos were returned to M16 medium at 37 °C under 5% CO_2_ in air, and successfully fused embryos were cultured to blastocyst stage. Tetraploid complementation was carried out by injecting 10–15 epigenome-edited ESCs (C57BL/6 J x 129X1/svJJmsSlc) or control wild-type ESCs into the blastocoel cavity of tetraploid blastocysts using the Piezo Micro Manipulator (Prime Tech, Ibaraki, Japan). The injected blastocysts were transferred to the uterine horns of pseudopregnant ICR females at 2.5 days post coitus.

### Microinjection of zygotes

Microinjection was conducted at 24–27 h post hCG as previously reported [53]. For transient expression of epigenome editing factors in zygotes, in vitro transcribed dCas9-5xPlat2 mRNA (50 ng/μl), scFvGCN4sfGFPTET1CD mRNA (75 ng/μl), and nine gRNAs for *H19*-DMR (5 ng/μl each) and optionally four gRNAs for *H19*-promoter (5 ng/μl each) were injected into the cytoplasm of fertilized embryos in M2 medium. For stable expression of epigenome editing factors, linearized all-in-one vector was inserted into the *Rosa26* locus. In brief, all-in-one vector with *H19* gRNAs (pPlatTET-gRNA2, 35 ng/μl), AsCpf1 protein (100 ng/μl; A.s. Cpf1 Nuclease 2NLS, #1076152, IDT), and *Rosa26* gRNA for Cpf1(14 ng/μl; Additional file 1: Table S4) were injected into the pronuclei of fertilized eggs. The injected embryos were cultured in M16 medium at 37 °C under 5% CO_2_ in air. Two-cell stage embryos were transferred into the ampulla of the oviduct (20–25 embryos per oviduct) of pseudopregnant ICR females. For the vector integration analysis, the genomic DNAs were extracted from tail tips of mice using a DNA extraction kit (DirectPCR Lysis Reagent, Mouse Tail; Viagenbiotech, CA). PCR was performed using the primer set (Additional file 1: Table S4).

### DNA methylation analysis

Genomic DNA was extracted from cultured cells, whole bodies of newborn mice, and livers of adult mice at 8 weeks of age using the AllPrep DNA/RNA Mini Kit (QIAGEN). Extracted DNA was treated with the Epitect Plus DNA Bisulfite Kit (QIAGEN) according to the manufacturer’s instruction. The modified DNA was amplified with the PCR primers described in Additional file 1: Table S4 and S5. The percentages of demethylation at CpG sites were determined by COBRA. Briefly, amplified fragments were cleaved with restriction enzymes (Additional file 1: Table S4 and S5) whose recognition sites were located in these sites and subjected to PAGE. Methylation level was calculated as the percentage of cleaved DNA/total DNA by densitometric analysis of gels stained with ethidium bromide using ImageJ software (NIH). DNA methylation analysis of potential off-target sites and human cells was performed using capillary and microchip electrophoresis (MCE-202 MultiNA, Shimadzu, Kyoto, Japan).

### Quantitative RT-PCR analysis (qPCR)

Total RNA was prepared from isolated tissues using the AllPrep DNA/RNA Mini Kit (QIAGEN). Gene expression levels were measured with LightCycler 96 (Roche) using SYBR Premix Ex Taq (TakaRa) according to the manufacturer’s instructions. Expression levels were normalized against the level of *Gapdh* (mouse) or *ACTB* (human). Primer sequences are described in Additional file 1: Table S4 and S5.

### Chromatin immunoprecipitation

Chromatin immunoprecipitation (ChIP) was performed as previously described with some modifications [54]. Cells were fixed by 1% formaldehyde. After being washed by PBS, lysates were suspended in cell lysis buffer (5 mM PIPES pH 8.0, 85 mM KCl, 0.5% NP-40), and incubated on ice for 15 min. Lysates were then homogenized using Dounce homogenizer with a loose pestle. After centrifugation, pellets were resuspended in SDS-lysis buffer (50 mM Tris-HCl pH 8.0, 10 mM EDTA, 1% SDS) and subjected to chromatin fragmentation using a Picoruptor sonicator (Diagenode, Liege, Belgium). Soluble chromatin fraction was incubated with anti-CTCF antibody (Millipore, 07-729) or normal rabbit IgG (Santa Cruz, sc-2027) at 4 °C overnight and then pulled down by Protein A/G Agarose Beads (Millipore). Purified DNA was subjected to quantitative PCR using the primers listed in Additional file 1: Table S4 and S5.

### Bisulfite amplicon sequencing analysis

For comprehensive CpG methylation analysis around *H19* gene, genomic DNA treated with the Epitect Plus DNA Bisulfite Kit (QIAGEN) were amplified using 24 primer pairs for each sample (Additional file 1: Table S4). The pooled PCR products were used for the preparation of a fragment library by the NEBNext Ultra II DNA Library Prep Kit for Illumina (NEB). Briefly, 500 ng of PCR product was sheared using the Covaris® S220 System (Covaris Inc., MA) for 150 s with a duty cycle of 10%, 2 cycles, 200 cycles/burst at 5 °C. The adaptors were ligated to both ends of the DNA to generate a fragment library by the NEBNext Adaptor (NEB). The barcoded libraries were amplified by PCR for 5 cycles using the NEBNext Multiplex Oligos for Illumina (NEB). The prepared libraries were then sequenced on the Illumina MiniSeq (Illumina, San Diego, CA). Generated raw sequence data in FASTQ format were imported into CLC Genomics Workbench 12.0.3 (QIAGEN), trimmed using Trim reads 2.3 tool, and mapped to the 15-kb regions of reference sequence (NC_000073, *Mus musculus* strain C57BL/6J chromosome 7, GRCm38.p6 C57BL/6J) using Map Reads to Reference 1.6 tool. For CpG methylation analysis, trimmed libraries were mapped to the reference sequence described above using Map Bisulfite Reads to Reference tool, and 5-mC percentages were calculated using Call Methylation levels 1.1 tool.

### Micro-CT and 3D reconstruction

Mice at 4 weeks of age were scanned with a computed tomography (CT) machine (Latheta LCT-200, HITACHI, Tokyo, Japan). Three-dimensional cross sections were generated with a resolution of one cross section per 96 μm. The images were transformed into DICOM format, and landmarks were placed within the 3D representation at the endpoints of craniofacial bones and limb bones in the forelimb (humerus and ulna bones) and hindlimb (femur and tibia bones) using VGStudio MAX2.2 (Volume Graphics, Heidelberg, Germany). Linear measurements were obtained for each pair of landmarks.

### Statistical analysis

Data are shown as means and standard deviations. The Student’s *t* test (two-tailed test) was used for DNA methylation, gene expression, and body weight analyses. One-way ANOVA was used for ChIP-qPCR analysis. To analyze the correlations in these variables, Pearson’s correlation coefficients (*r*) were calculated. A *P* value of < 0.05 was considered significant. No sample size estimates were performed.

## Supplementary information

Additional file 1: Table S1. Generation of EpiEdit mice derived from ESCs *via* tetraploid complementation. **Table S2.** Generation of EpiEdit mice by transient expression of epigenome editing factors. **Table S3.** Generation of EpiEdit mice by stable expression of epigenome editing factors. **Table S4.** gRNAs and primers for mouse experiment. **Table S5.** gRNAs and primers for human experiment.

Additional file 2: Figure S1. GFP intensity correlate with DNA demethylation levels. **Figure S2.** Expression of GFP in preimplantation embryos that transiently expressed epigenome editing factors. **Figure S3.** Expression of epigenome editing factors in preimplantation embryos and newborn mice that stably expressed epigenome editing factors. **Figure S4.** DNA methylation and gene expression in epigenome-edited mice. **Figure S5.** Confirmation of vector integration. **Figure S6.** Targeted demethylation in *H19*-DMR increases CTCF-binding. **Figure S7.** DNA methylation analysis for potential off-target regions by COBRA. **Figure S8.** Other phenotypes of vector-integrated (*H19*-DMR) epigenome-edited mice. **Figure S9.** Germline transmission capability of a vector integrated mouse. **Figure S10.** Plasmid map of pPlatTET-gRNA2-H19DMRx9 all-in one vector. **Figure S11.** Plasmid map of pPlatTET-gRNA2-H19P (H19 promoter) all-in one vector. **Figure S12.** Plasmid map of pPlatTET-gRNA2 all-in one vector.

Additional file 3: Review history.

## Data Availability

All amplicon sequencing data have been deposited with links to DRA accession number DRA009751 in the DDBJ database (https://www.ddbj.nig.ac.jp/dra/index-e.html) [55].
