# Supplementary material for: Successful generation of epigenetic disease model mice by targeted demethylation of the epigenome
Source: Genome Biol. 2020 Apr 1;21:77. doi: 10.1186/s13059-020-01991-8 (PMC7110793; doi:10.1186/s13059-020-01991-8)
Supplement: Supplementary file 2 — GFP intensity correlate with DNA demethylation levels. Figure S2. Expression of GFP in preimplantation embryos that transiently expressed epigenome editing factors. Figure S3. Expression of epigenome editing factors in preimplantation embryos and newborn mice that stably expressed epigenome editing factors. Figure S4. DNA methylation and gene expression in epigenome-edited mice. Figure S5. Confirmation of vector integration. Figure S6. Targeted demethylation in H19-DMR increases CTCF-binding. Figure S7. DNA methylation analysis for potential off-target regions by COBRA. Figure S8. Other phenotypes of vector-integrated (H19-DMR) epigenome-edited mice. Figure S9. Germline transmission capability of a vector integrated mouse. Figure S10. Plasmid map of pPlatTET-gRNA2-H19DMRx9 all-in one vector. Figure S11. Plasmid map of pPlatTET-gRNA2-H19P (H19 promoter) all-in one vector. Figure S12. Plasmid map of pPlatTET-gRNA2 all-in one vector. [file 13059_2020_1991_MOESM2_ESM.pdf]

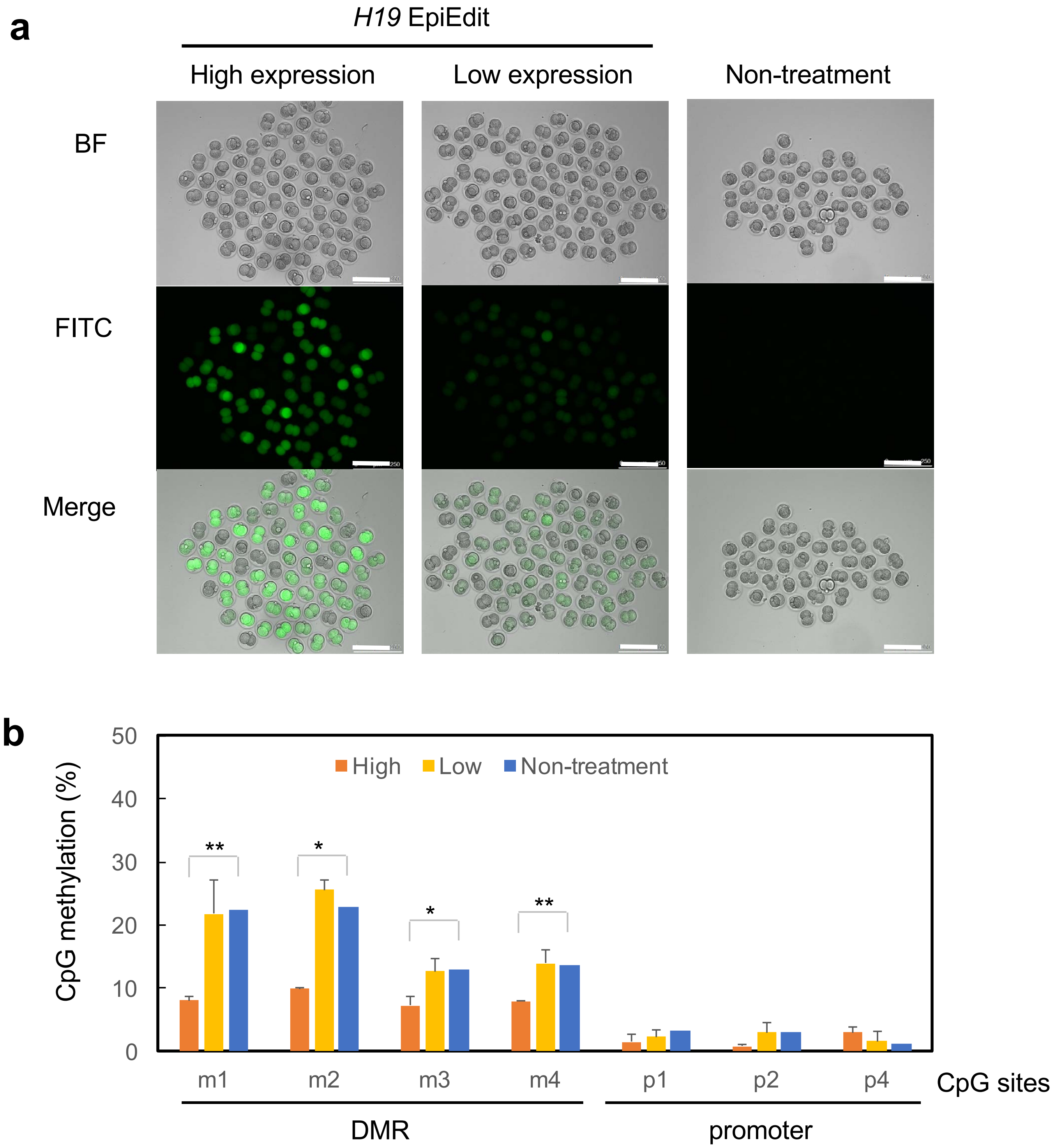

Fig. S1

GFP intensity correlate with DNA demethylation levels.

(a) RNA for *H19* EpiEdit (DMR+promoter) with high (135 ng/μl) or low (27 ng/μl) concentration were injected to the cytoplasm of fertilized eggs. GFP intensity at 2-cell stage depend on the concentration of injected RNA. **(b)** DNA methylation analysis (COBRA) of *H19*-DMR and promoter using pooled blastocysts (n=40, respectively) shows strong correlation between GFP intensity and levels of demethylation. bars, mean ± s.d. * $P < 0.05$, n.s., not significant (two-tailed Student's *t-test).* Scale bars, 200 μm.

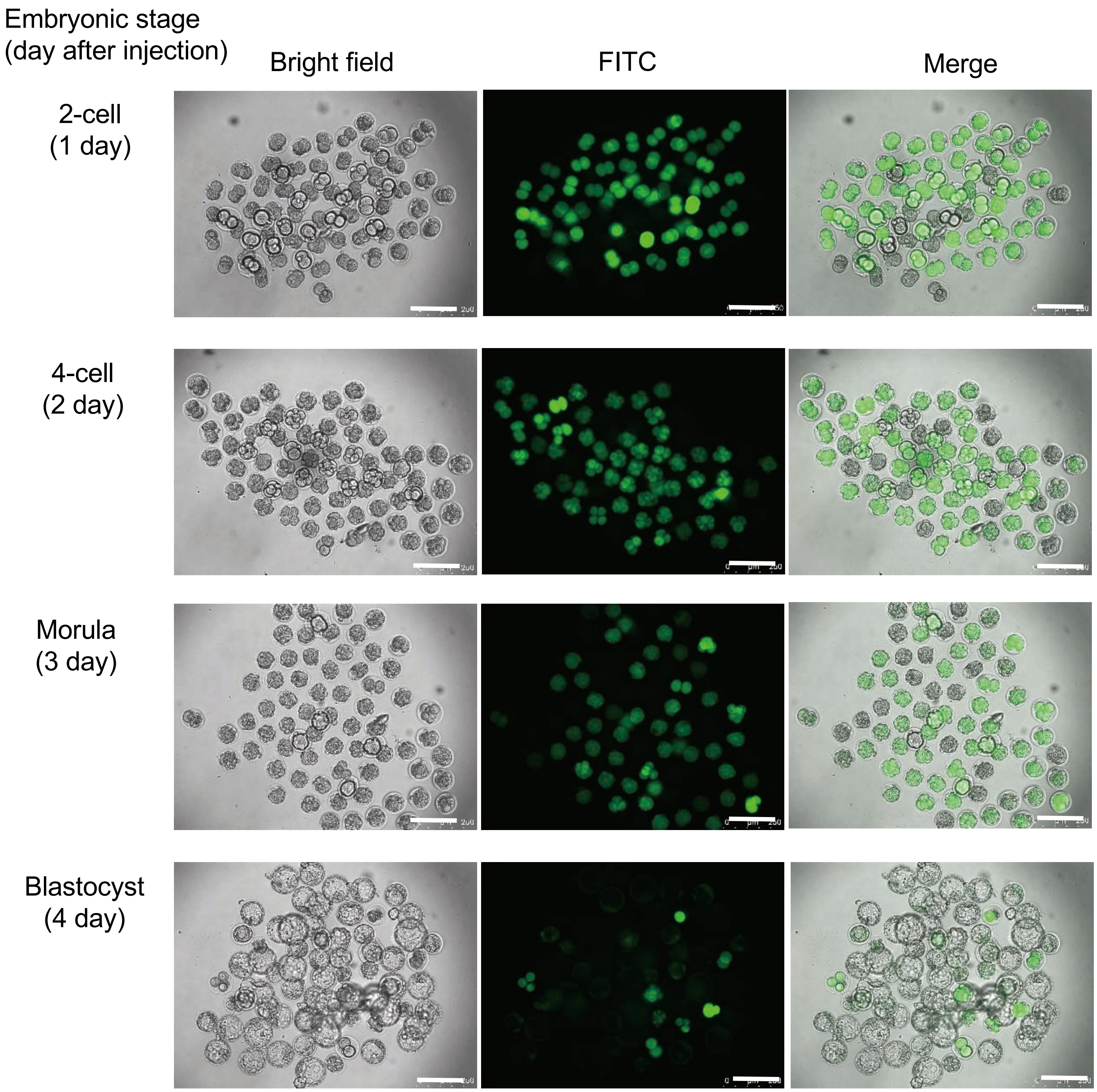

Fig. S2

Expression of GFP in preimplantation embryos that transiently expressed epigenome editing factors. Strong GFP expression was observed from the 2-cell to morula stage. However, the signal had mostly disappeared at the blastocyst stage except for those embryos whose development had stopped. Scale bars, 200 μm.

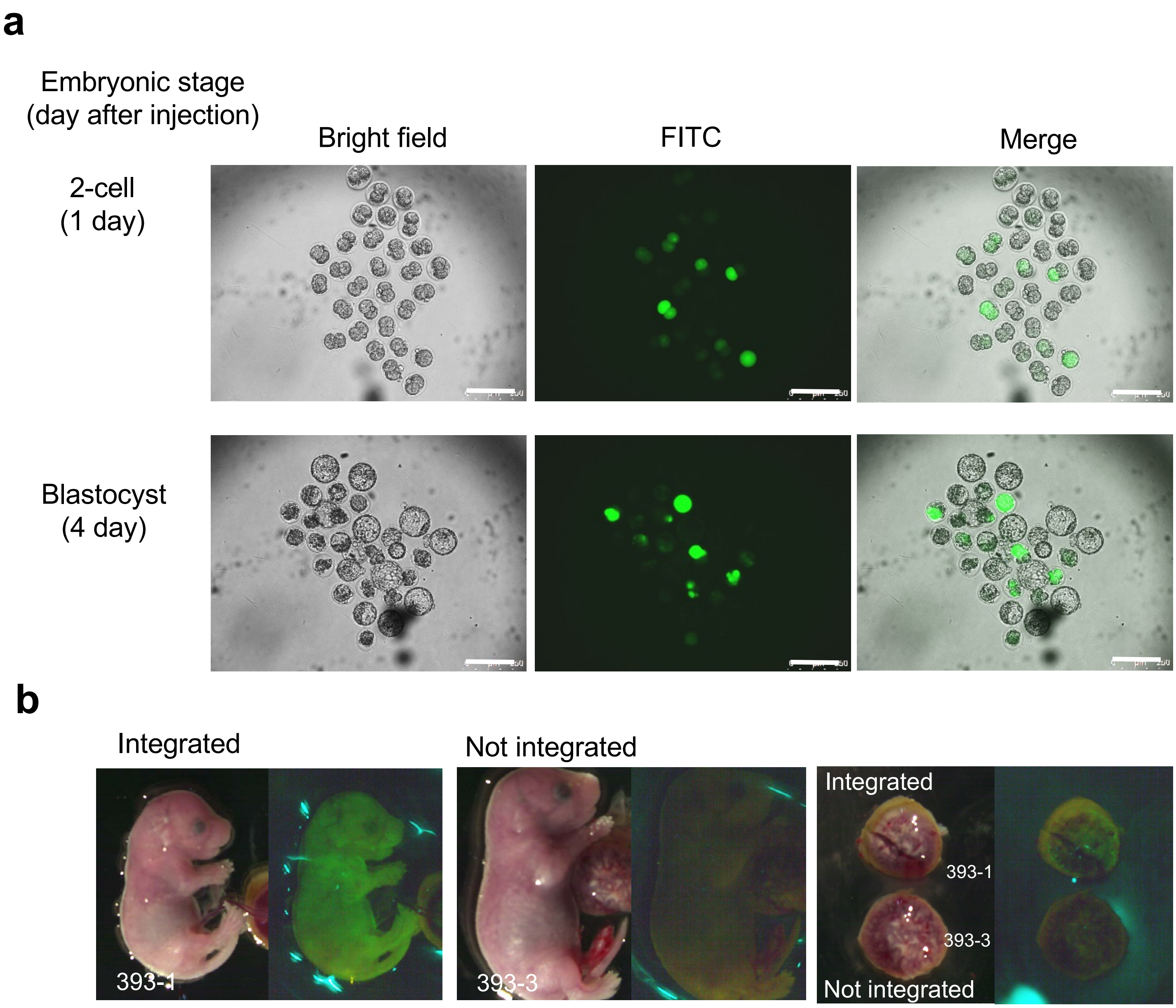

Fig. S3

Expression of epigenome editing factors in preimplantation embryos and newborn mice that stably expressed epigenome editing factors. Expression of GFP in (**a**) preimplantation embryos and (**b**) newborn mice and placentas that stably expressed epigenome editing factors for *H19*-DMR. Scale bars, 200 μm.

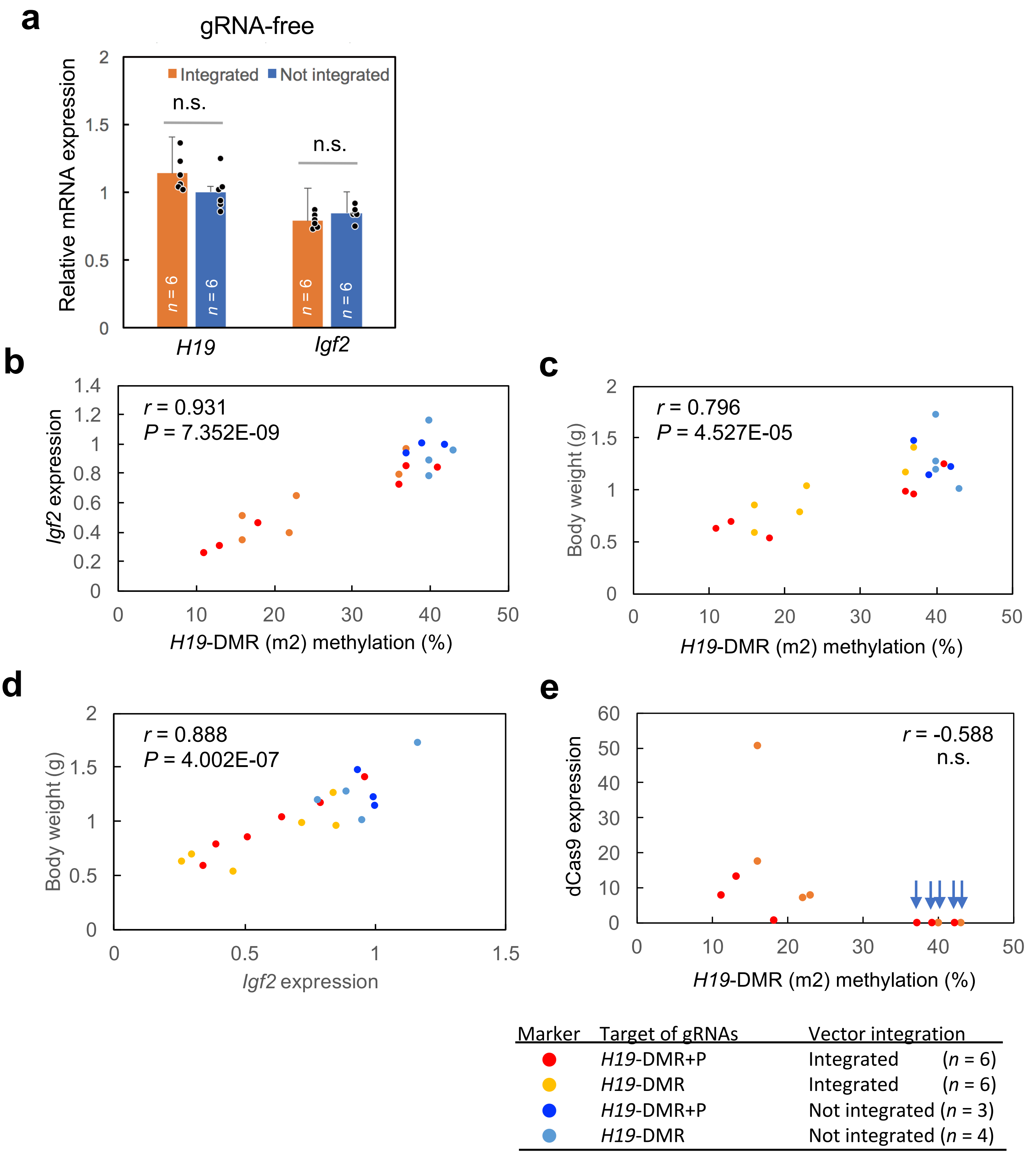

Fig. S4

DNA methylation and gene expression in epigenome-edited mice.

(**a**) Expression analysis by qPCR. gRNA-free vector-integrated newborn mice did not show any significance in *H19* and *Igf2* expression. *n* indicates the number of mice used for analysis. Error bars, mean ± s.d. (samples from two independent experiments). n.s., not significant (two-tailed Student's *t-test).* **(b-d)** In the mice generated by the stable expression of epigenome editing factors, correlations among *H19*-DMR methylation (m2 site), *Igf2* expression, and body weight were measured by Pearson's correlation coefficient (*r*). Strong correlations were found among these parameters. **(e)** Vector-integrated mice without dCas9 expression did not show methylation change in *H19*-DMR (arrows). * *P* < 0.05, n.s., not significant (two-tailed Student's *t-test)*

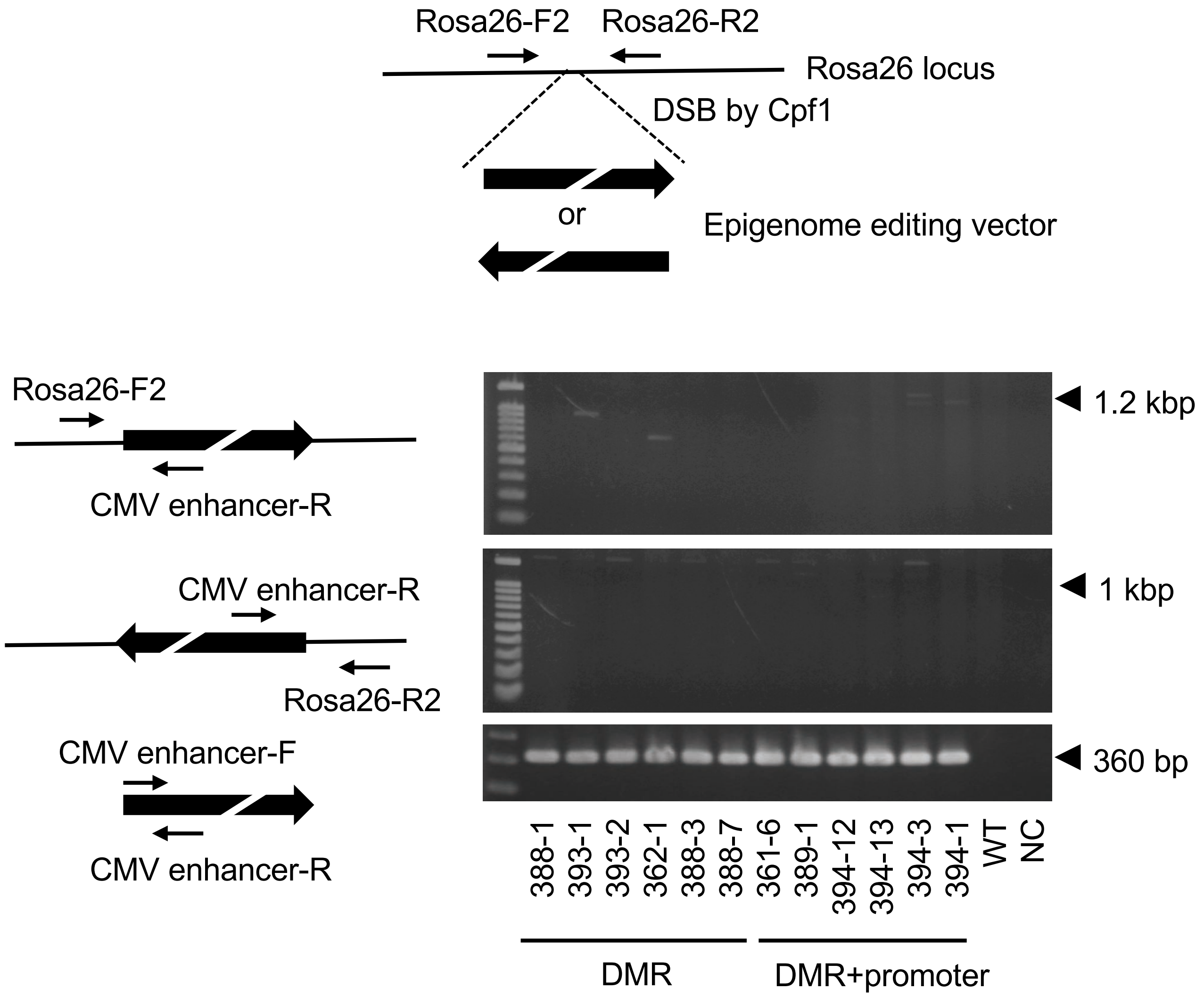

Fig. S5

Confirmation of vector integration.

We attempted to introduce the epigenome editing vector (18 kbp) into the Rosa26 locus by NHEJ; however, PCR analysis clarified that the vector was not integrated into the Rosa26 locus but other loci. WT, wild type control; NC, non-template control.

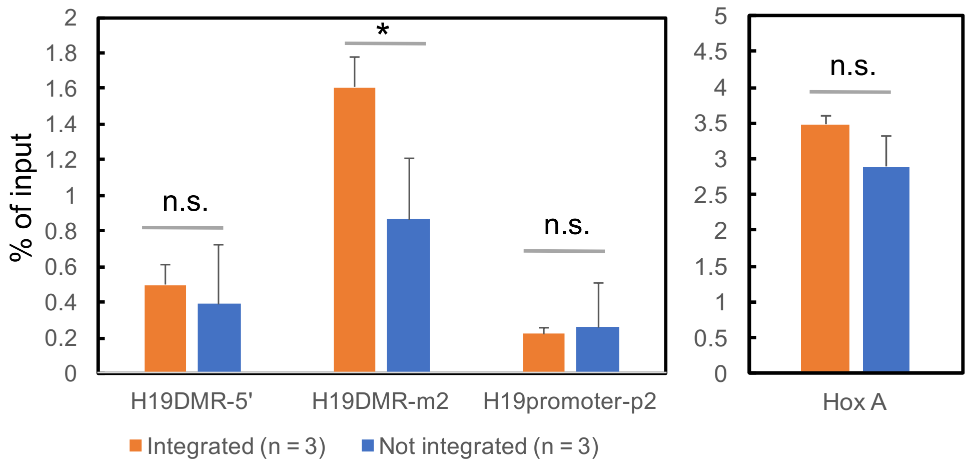

Fig. S6

Targeted demethylation in *H19*-DMR increases CTCF-binding.

Anti-CTCF ChIP was performed using cells in a followed by qPCR analysis. Targeting demethylation in *H19*-DMR increases CTCF-binding to the m2 site. Upstream (H19DMR-5') and downstream (H19promoter-p2) of *H19*-DMR was selected as negative control of ChIP-qPCR. The CTCF binding site within Hox A gene cluster was selected as positive control. *n* indicates the number of mice used for ChIP-qPCR. bars, mean ± s.d. * $P < 0.05$, n.s., not significant (one-way ANOVA)

Mismatch base: 2
H19DMR_3 OT
AAATCTGTTATGTGCAACAAGGG

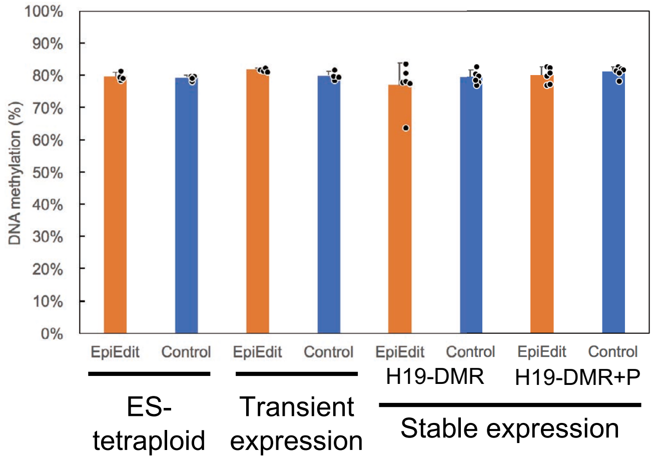

Mismatch base: 2
H19DMR_11 OT
CCCCCCAGCCATAAAAGCAGAGG

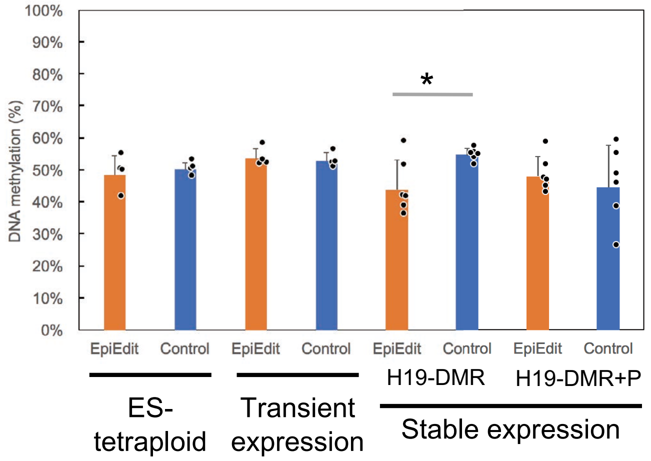

Mismatch base: 2
H19DMR_10 OT2
AGGGAGGGAGTCAAGTTCTCTGG

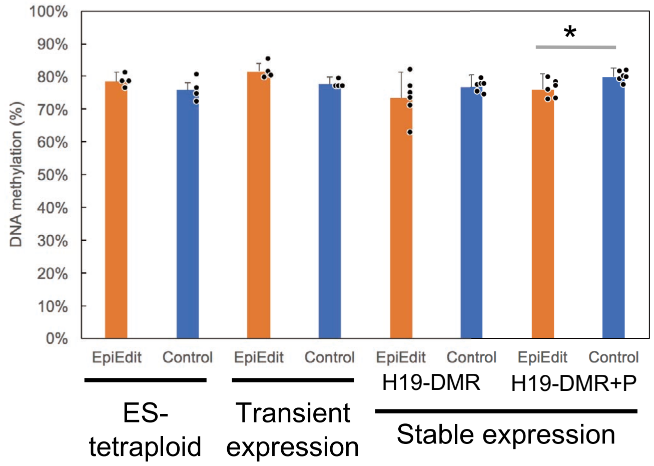

Mismatch base: 3
H19DMR_10 OT
TGAAAATGAGTCAAGTTCTCTGG

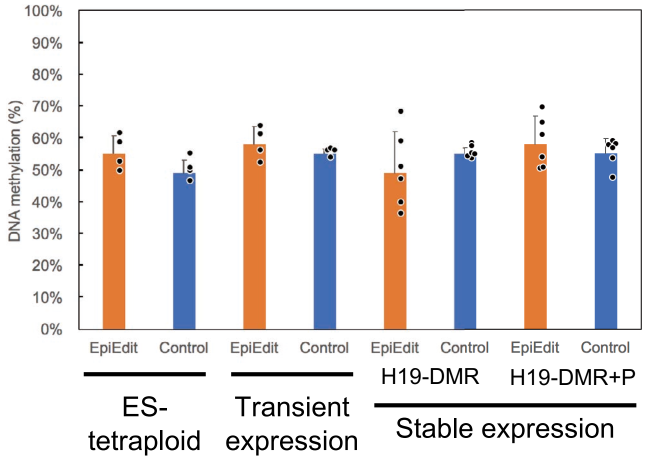

Mismatch base: 3
H19DMR_7 OT3
GTTTCTAAGTGTGGTGATCAGGG

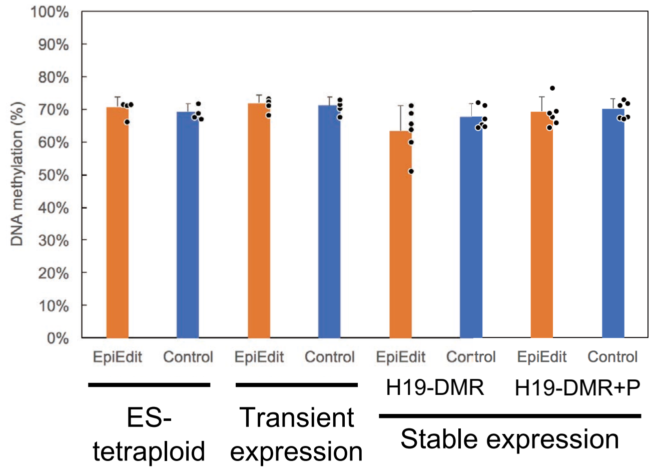

Mismatch base: 3
H19P3 OT
ACAGGTTAGTTGACAGAGTAAGG

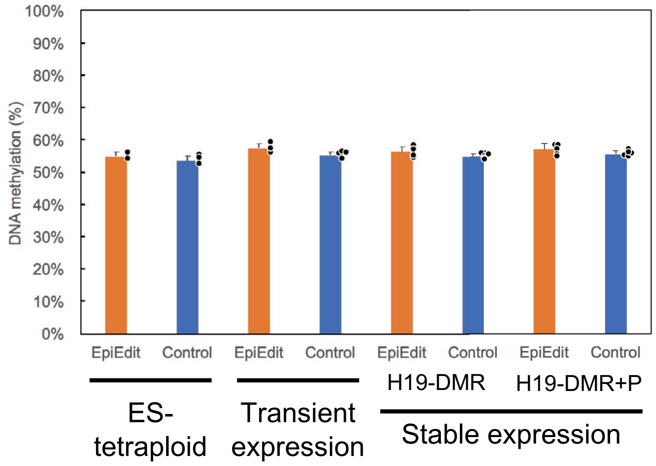

Fig. S7

Continued…

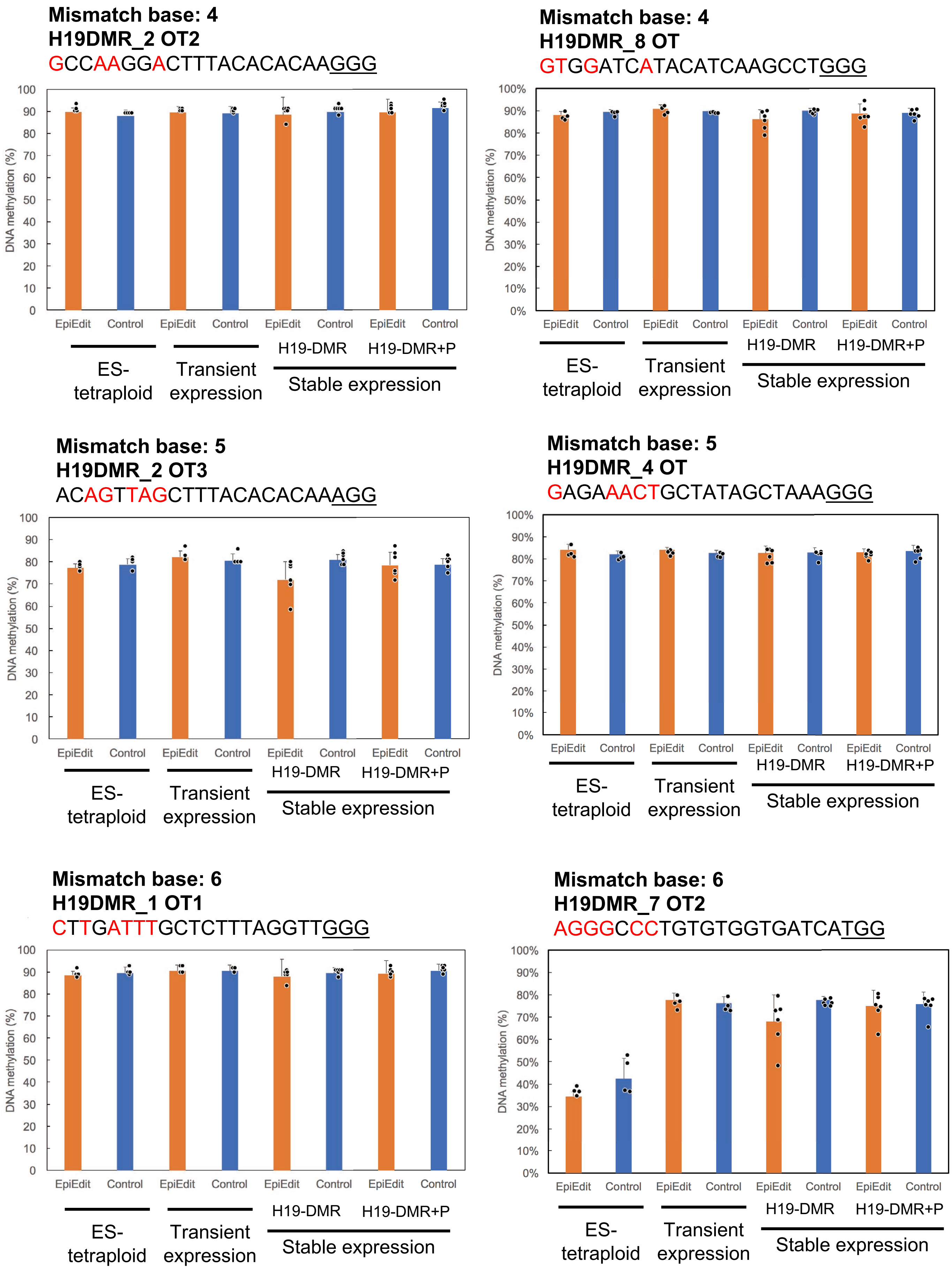

Fig. S7

DNA methylation analysis for potential off-target regions by COBRA .

DNA methylation status for potential off-target regions that exactly matches 12 bases at the 3' end and the NGG (PAM) was examined in newborn mice. In particular, all sites with less than 4 mismatches (6 regions), and each 2 potential off-target regions with 4, 5 and 6 mismatches (6 regions) were analyzed by COBRA. bars, mean ± s.d. * $P < 0.05$ (two-tailed Student's *t*-test). Mismatched base (highlighted), PAM site (underlined).

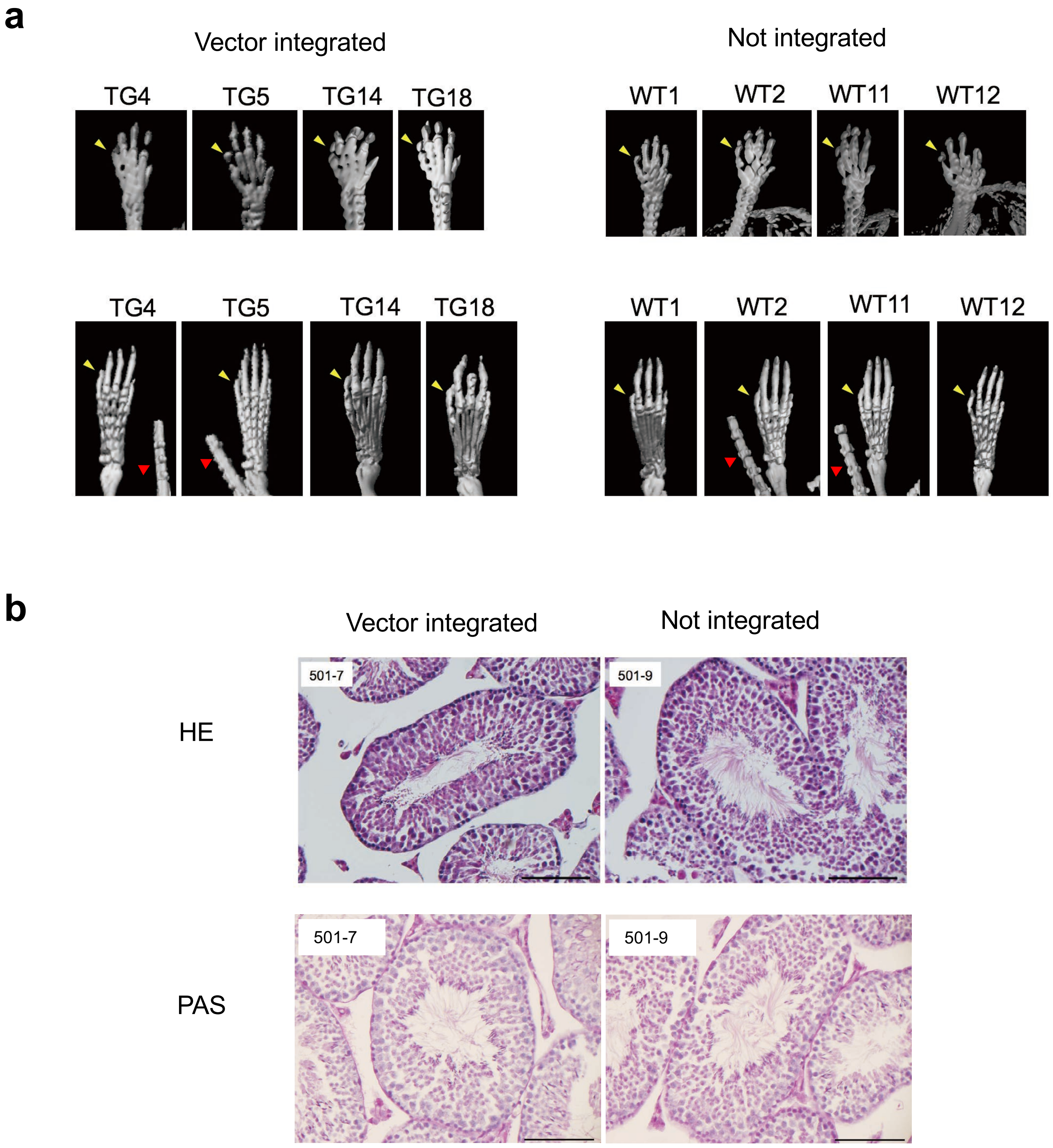

Fig. S8

Other phenotypes of vector-integrated (*H19*-DMR) epigenome-edited mice.

(a) 3D bone image of forepaw (*upper*) and hind paw (*lower*) reconstructed from CT scan of a mouse. Epigenome-edited mice did not show apparent clinodactyly of the fifth finger (yellow arrow head) as shown in SRS patients. Red arrow head indicates tail. TG18 mouse accidentally lost the middle finger in the left hind paw.

(b) Hematoxylin and eosin (HE) and Periodic acid-Schiff (PAS) -stained section of seminiferous tubules (stage VIII) at 8 weeks. Normal spermatogenesis was observed in the testis of vector-integrated epigenome-edited mice. Scale bars, 100 μm.

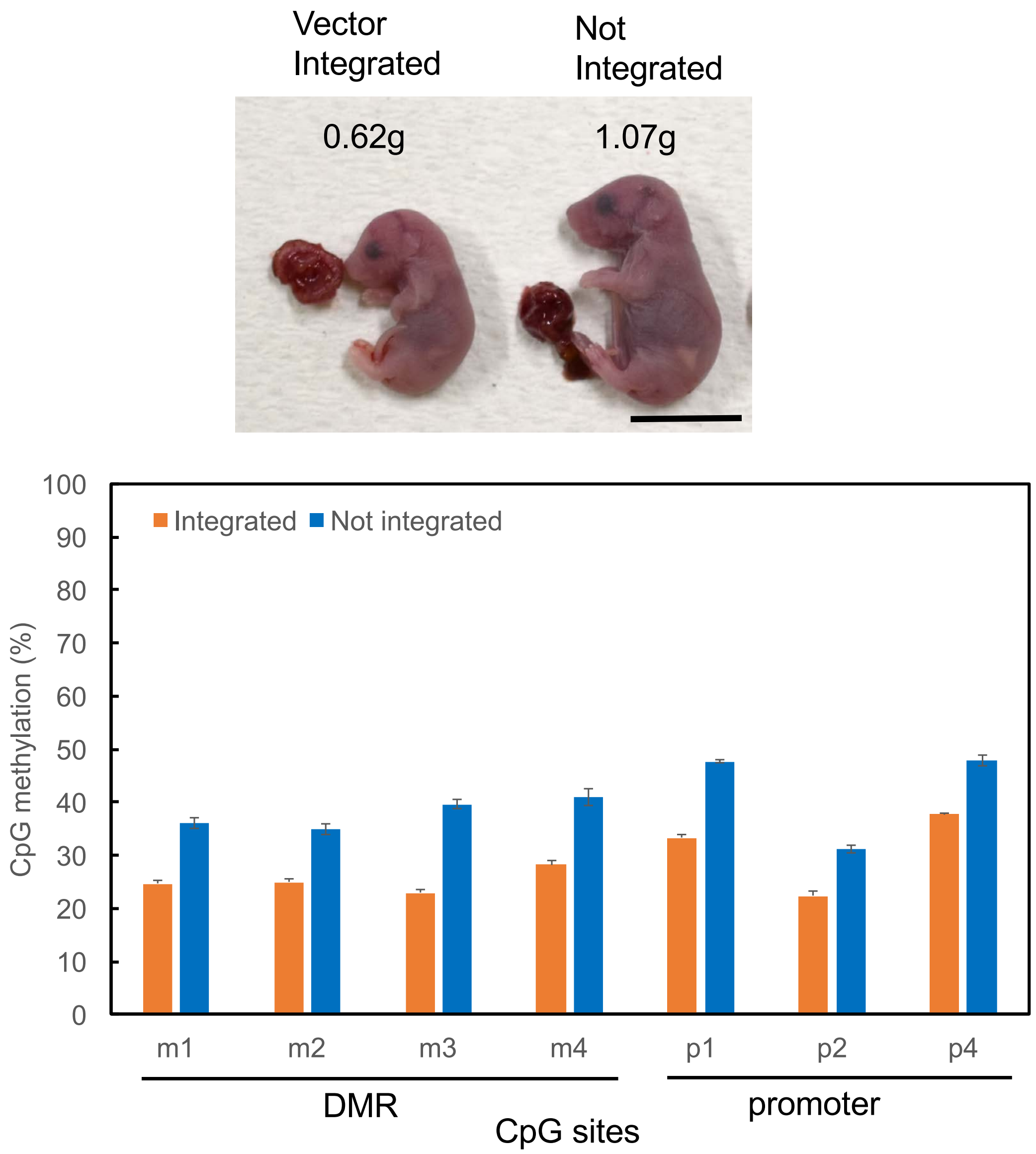

Fig. S9

Germline transmission capability of a vector integrated mouse.

CpG methylation status in *H19*-DMR and promoter was analyzed by COBRA using F1 offspring (e18.5) derived from a vector-integrated female founder mouse (TG20). Apparent demethylation was observed in vector-integrated F1 mice. bars, mean ± s.d. Scale bars, 1 cm.

pPlatTET-gRNA2-H19DMRx9

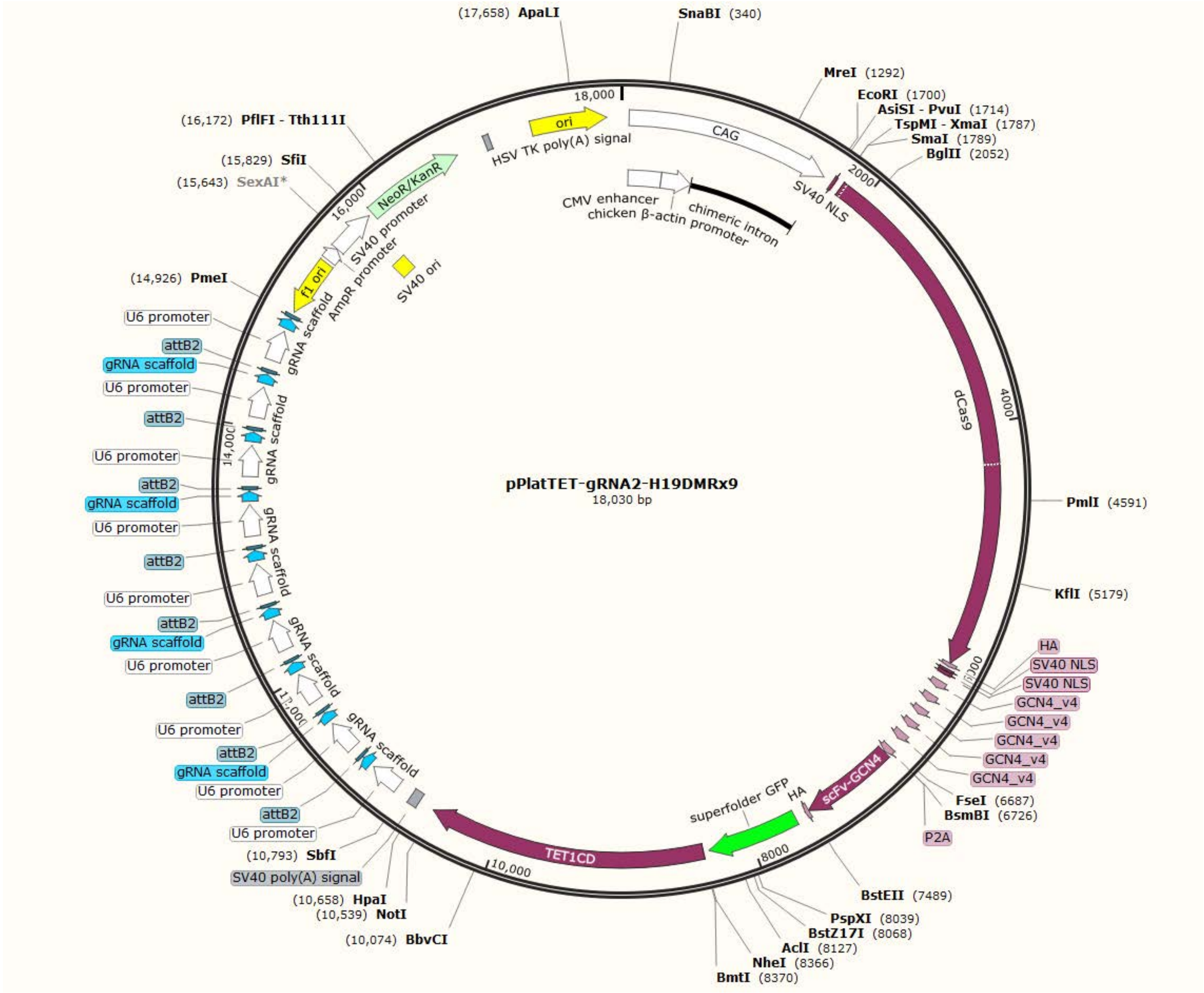

Fig. S10
Plasmid map of pPlatTET-gRNA2-H19DMRx9 all-in one vector.

pPlatTET-gRNA2-H19P

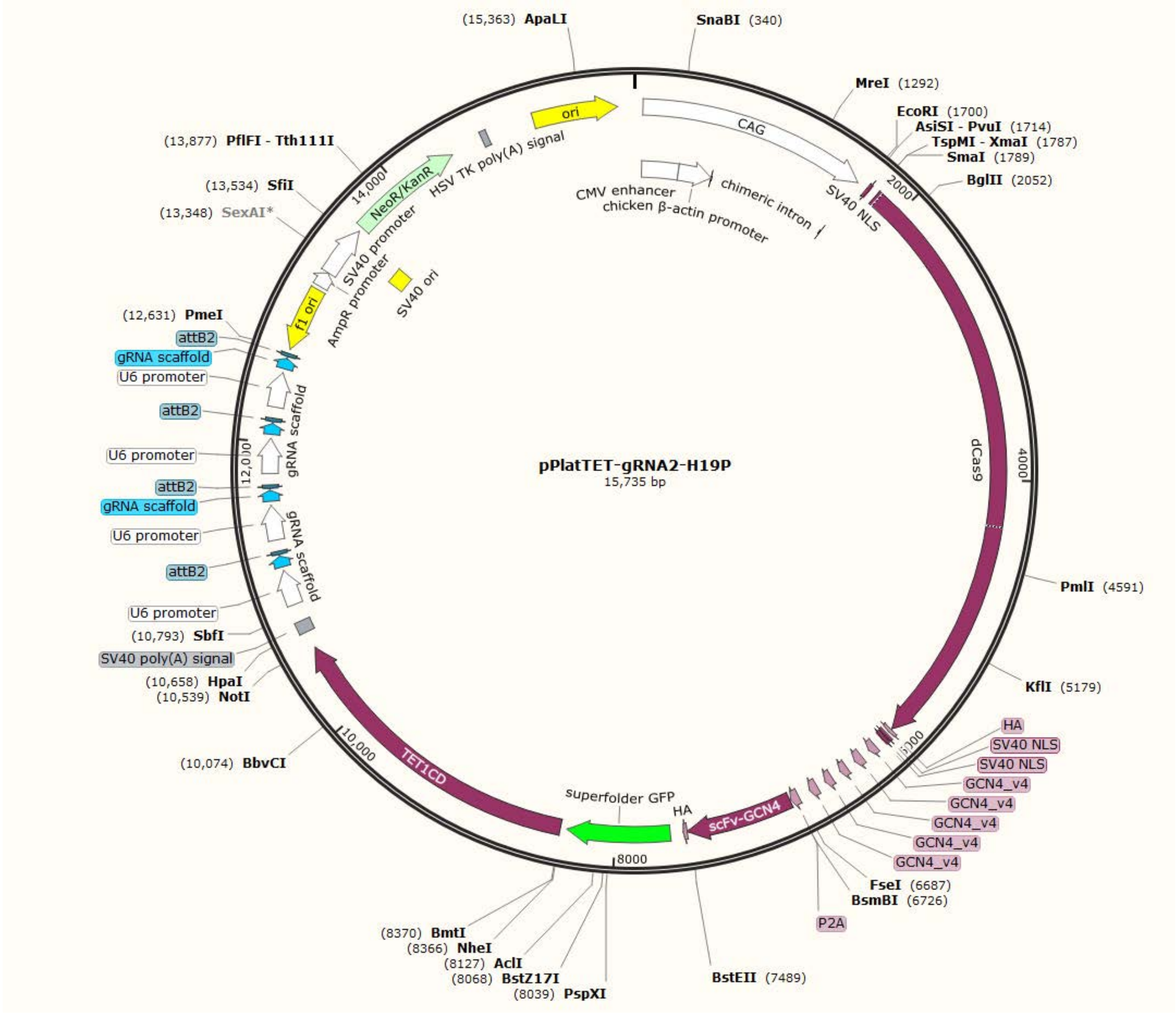

Fig. S11
Plasmid map of pPlatTET-gRNA2-H19P (*H19* promoter) all-in one vector.

pPlatTET-gRNA2

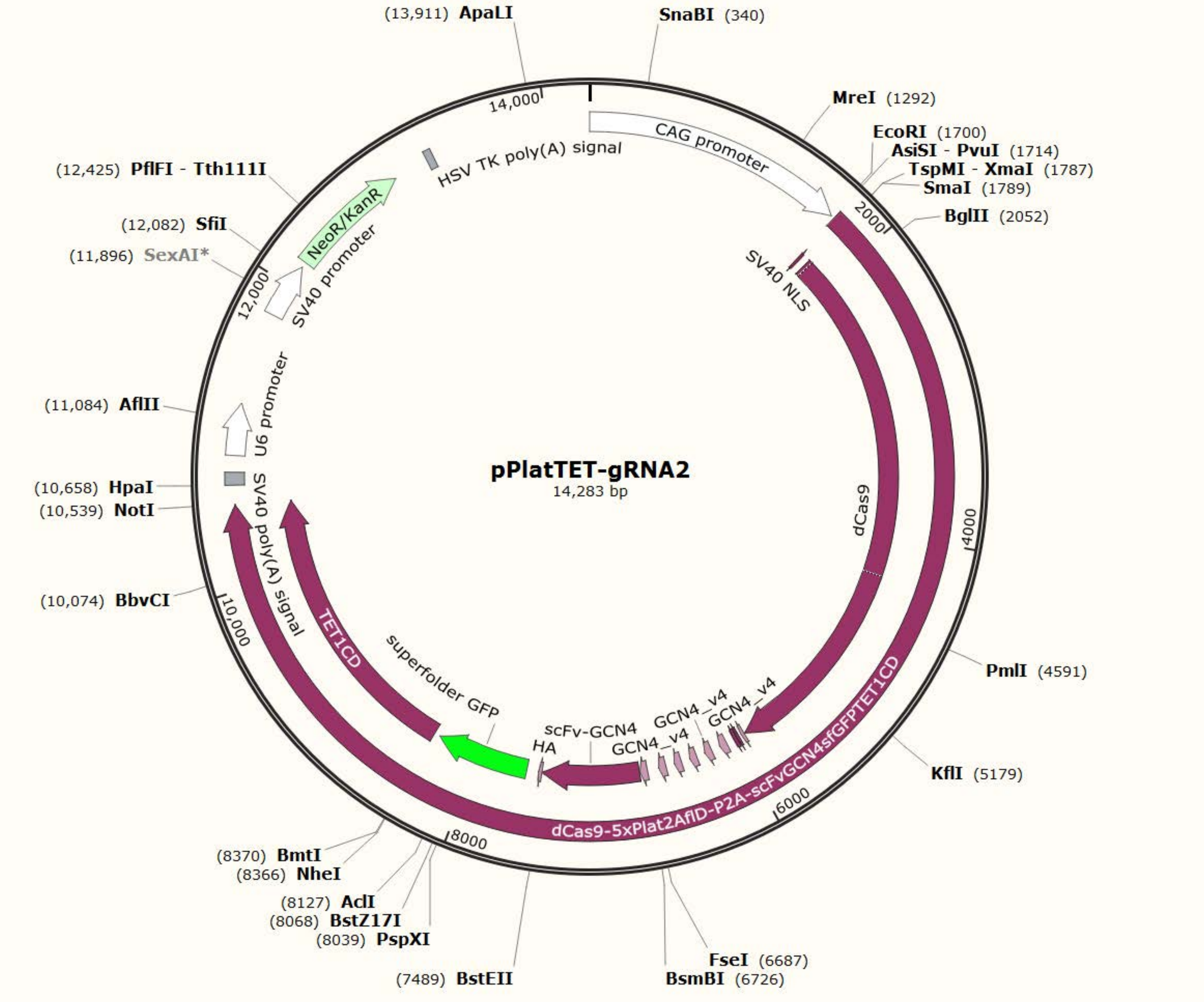

Fig. S12
Plasmid map of pPlatTET-gRNA2 all-in one vector.
