# Supplementary material for: Successful generation of epigenetic disease model mice by targeted demethylation of the epigenome
Source: Genome Biol. 2020 Apr 1;21:77. doi: 10.1186/s13059-020-01991-8 (PMC7110793; doi:10.1186/s13059-020-01991-8)
Supplement: Supplementary file 3 — Review history. [file 13059_2020_1991_MOESM3_ESM.docx]

Review History

**First round of review**

**Reviewer 1**

**Are you able to assess all statistics in the manuscript, including the appropriateness of statistical tests used?**

Yes, and I have assessed the statistics in my report.

**Comments to author:**

The study by Horii et al entitled "Successful generation of epigenetic disease model mice by targeted demethylation of the epigenome" compares three different methods to generate mice specifically demethylated at the imprinted H19-DMR locus (referred to as epi-edited mice). Demethylation is accomplished by targeting the dCas9-Suntag system and TET1 to the H19-DMR locus. The authors look into the feasibility of using epi-edited mice as a SRS disease model by looking at RNA expression, methylation and SRS body weight phenotype. Transient exposure to the editing tools causes methylation changes in derived epi-edited mice, but only stable integration and constant expression of epi-editing tools also show an effect on body weight, a SRS phenotype in human disease. Overall this is a well written and interesting study that would be of interest to the field. However, further simple experiments (using already collected materials) could help to draw stronger conclusions of variable effects by individual methods.

Major comments
Three methods are compared, but the methylation sites analyzed vary between experiments (m1-m4 for method 1, only m2 for method 2, m1-m4 and p1-p4 for method 3). Methylation analysis should be carried out for m1-m4 and p1-p4 for all three methods. Spreading of de-methylation might be another criteria for the method-specific effects the authors see. I was struck by the demethylation occurring over a 7-8kb region in Suppl. Fig 4b. It would be interesting to see how other methods compare.

I am not convinced that demethylation was successful using method 2. Fig 3d identifies 4 mice (of 35 edited mice) with demethylated H19-DMR. However, 35% methylation was chosen as an arbitrary cut-off. Were statistical methods used to choose this cut-off? Demethylation in these four mice is not convincing. Maybe this method was simply not successful? Again, I think that looking at spreading of demethylation over more than one site would be very informative.

Method 3 is based on stable integration of epigenetic editing tools and the data are very convincing. However, constant overexpression and targeting of TET1 could certainly lead to a variety of off-target demethylation. To ensure that the weight phenotype observed is only due to H19-DMR demethylation, a broader off-target analysis should be performed. 9 gRNAs are used simultaneously (sometimes 13 gRNAs), which all may have potential off-targets. Only 3 potential off-targets were investigated with 4, 5 and 7 mismatches each. Surely there are regions in the genome with less than 4 mismatches for some of the gRNAs. This should be investigated more thoroughly.


Minor comments
It would be helpful to show the methylation status of the H19 promoter in addition to the H19-DMR either in Figure 1 or the text.

Page 44/Line 677: TET1CD initiates demethylation (TET1 does not demethylate per se)

Fig2c: gRNA-free should be changed to "Non-treatment" to be consistent with other figure panels.

Methylation analysis in newborns was done on the entire pup?

Some figures need to be tidied up and neatly aligned. It seems that some legends, scale bars, etc. shifted (perhaps during pdf conversion).

Methods 1&2 do not yet seem an efficient way to generate epi-edited mice. How would the authors propose to improve on this considering that the epigenetic modification will not be passed on to the next generation?

Although stable integration occurs in the same locus (Rosa26) for all epi-edited mice, there is variation in dCas9 expression. Ca

**Reviewer 2**

**Are you able to assess all statistics in the manuscript, including the appropriateness of statistical tests used?**

No, I do not feel adequately qualified to assess the statistics.

**Comments to author:**

In this manuscript, Horii et al generate a mouse model for an imprinting disorder, Silver-Russel Syndrome, by targeted demethylation of the H19 differentially methylated region (H19-DMR). The authors use a dCas9-SunTag and scFv-GFP-TET1 based system to perform the epigenome editing by 3 different methods using mouse embryonic stem cells or fertilized eggs. In the first method, the authors perform tetraploid complementation that enables generation of mice derived entirely from epigenome edited ES cells. In the second method, the authors overcome the limitation of absence of ESC lines in non-rodent animals by transiently expressing the mRNA for epigenome editing in fertilized eggs to generate epigenome edited mice. A major limitation of both the above methods is that the epigenome editing does not undergo germline transmission. In the third method, the authors overcome this hurdle by stably expressing the epigenome editing factors by targeting them to the Rosa26 locus. Additionally, the authors have demonstrated that their method is also effective in human cells by replicating their experiment in HEK293 cells.

Major points:
1.     The authors have performed all the DNA methylation analysis using COBRA. COBRA detects the methylation status at the recognition sites of the restriction enzymes, and therefore is not the best tool to assay for DNA methylation in this study. Because the entire paper is based on the epigenome editing, it is imperative for the authors to validate their experiments using a method that comprehensively determines the DNA methylation across the entirety of H19 promoter and H19 differentially methylated region. Ideally, the authors would use a sequencing-based approach such as amplicon sequencing to validate their demethylation experiments.

2.     Apart from measuring the body weight, blood glucose level and cardiac fibrosis the authors do not perform a thorough phenotypic analysis of the mice obtained through each of the 3 methods. Because the objective of this paper is to generate a mouse model for an imprinting disorder, the authors should perform a deeper phenotypic analysis to show the amount of phenotypic overlap between their model and patients with SRS. The authors could qualify their claims for the model, and/or determine the Incidence of some of the additional phenotypes seen in patients such as macrocephaly, body asymmetry, feeding difficulties, clinodactyly, learning difficulties, and maxillofacial abnormalities.

3.     In Figs 2b, 3c, 3d, and 5c the methylation level at only one of the 4 CpG sites (m2) has been determined. Is there a reason why only one of the sites was chosen for the methylation analysis? The methylation level of m2 site might not accurately reflect the methylation level across the entire H19-DMR. At the very least, testing the methylation levels in all the 4 sites is necessary for such a claim.

4.     The authors need to demonstrate germline transmission of the H19-DMR demethylation in mice which stably express the epigenome editing factors to validate their claim in discussion.

Minor points:
1.     In Fig 2d the authors have determined the methylation level of newborn mice at the 4 CpG sites. Have the authors investigated if this trend continues during the postnatal development of the mice?
2.     The level of overexpression of H19 and downregulation of Igf2 in the unedited mouse appears to be comparable to that of the epigenome edited mice. Could the authors comment on this apparent discrepancy?
3.     In Fig3c legend, it is mentioned that the graph represents methylation at m1 and m2 contradicting the text.
4.     In Fig 3d, how did the authors decide 35% methylation as a cutoff for delineating methylated and demethylated H19-DMR?
5.     In the discussion, vector-integrated mice not showing H19-DMR demethylation having no Cas9 expression is not shown in Supp Fig6e, as is stated, but is shown in Sup Figure 5e.

**Authors Response**

**Point-by-point responses to the reviewers’ comments:**

Reviewer #1: The study by Horii et al entitled "Successful generation of epigenetic disease model mice by targeted demethylation of the epigenome" compares three different methods to generate mice specifically demethylated at the imprinted H19-DMR locus (referred to as epi-edited mice). Demethylation is accomplished by targeting the dCas9-Suntag system and TET1 to the H19-DMR locus. The authors look into the feasibility of using epi-edited mice as a SRS disease model by looking at RNA expression, methylation and SRS body weight phenotype. Transient exposure to the editing tools causes methylation changes in derived epi-edited mice, but only stable integration and constant expression of epi-editing tools also show an effect on body weight, a SRS phenotype in human disease. Overall this is a well written and interesting study that would be of interest to the field. However, further simple experiments (using already collected materials) could help to draw stronger conclusions of variable effects by individual methods.

*Response: We greatly appreciate the reviewer’s insightful comments about our study, which have helped us to significantly improve our paper.*

Major comments
Comment: Three methods are compared, but the methylation sites analyzed vary between experiments (m1-m4 for method 1, only m2 for method 2, m1-m4 and p1-p4 for method 3). Methylation analysis should be carried out for m1-m4 and p1-p4 for all three methods. Spreading of de-methylation might be another criteria for the method-specific effects the authors see. I was struck by the demethylation occurring over a 7-8kb region in Suppl. Fig 4b. It would be interesting to see how other methods compare.

*Response: We agree with your suggestion. Therefore, we have performed COBRA analysis for m1-m4 and p1-p4 for all samples generated by all three methods (Fig.2, Fig.3, and Fig.4). In addition, we have performed amplicon sequencing analysis which covers 145 CpG sites across 15kb regions around the H19-DMR for representative samples in all three methods (Fig. 5). We have added following descriptions in Results: “To compare comprehensive DNA methylation status among the three methods, amplicon sequencing analysis was performed for 145 CpG sites across 15 kb regions around H19-DMR (Fig. 5). H19-DMR, promoter and gene body regions were hypomethylated in all epigenome-edited newborn mice as expected. Among these samples, DNA methylation pattern was similar, and did not depend on gRNA (H19-DMR or H19-DMR+promoter) or method used (lines 204-209).”*

Comment: I am not convinced that demethylation was successful using method 2. Fig 3d identifies 4 mice (of 35 edited mice) with demethylated H19-DMR. However, 35% methylation was chosen as an arbitrary cut-off. Were statistical methods used to choose this cut-off? Demethylation in these four mice is not convincing. Maybe this method was simply not successful? Again, I think that looking at spreading of demethylation over more than one site would be very informative.

*Response: We sincerely apologize for any confusion. We decided cutoff between methylated and demethylated as the lowest methylation level (35%) in control mice at the m2 site in H19-DMR. In the revised manuscript, DNA methylation analysis was carried out for other 6 sites (m1, m3, m4, p1, p2 and p4). As a result, these 4 mice surely showed demethylation in each site compared to control mice (Fig. 3d arrows).*


Comment: Method 3 is based on stable integration of epigenetic editing tools and the data are very convincing. However, constant overexpression and targeting of TET1 could certainly lead to a variety of off-target demethylation. To ensure that the weight phenotype observed is only due to H19-DMR demethylation, a broader off-target analysis should be performed. 9 gRNAs are used simultaneously (sometimes 13 gRNAs), which all may have potential off-targets. Only 3 potential off-targets were investigated with 4, 5 and 7 mismatches each. Surely there are regions in the genome with less than 4 mismatches for some of the gRNAs. This should be investigated more thoroughly.

*Response: We thank the reviewer for this comment. We have performed DNA methylation analysis for all potential off-target regions (6 regions) in the genome with less than 4 mismatches for the 13 gRNAs. In addition, we have examined each 2 potential off-target regions with 4, 5 and 6 mismatches, respectively. As a result, we found demethylation in the two potential off-target regions for gRNA of H19DMR_10 and H19DMR_11 in the Method 3 sample, indicating that stable expression of epigenome editing factors could increase the risk of off-target epigenome editing (Sup Fig.7；lines 209-216).*

Minor comments
Comment: It would be helpful to show the methylation status of the H19 promoter in addition to the H19-DMR either in Figure 1 or the text.

*Response: Thank you for the helpful comment. We have shown the methylation status of the H19 promoter in the Fig.1a.*

Comment: Page 44/Line 677: TET1CD initiates demethylation (TET1 does not demethylate per se)

*Response: We thank the reviewer for this comment. This error has been corrected as follows: Thus, multiple copies of TET1CD hydroxylates specific loci and activates site-specific demethylation in the target (line 750).*

Comment: Fig2c: gRNA-free should be changed to "Non-treatment" to be consistent with other figure panels.

*Response: We thank the reviewer for this comment. This error has been corrected in accordance with the reviewer's comment (Fig. 2c).*

Comment: Methylation analysis in newborns was done on the entire pup?

*Response: Yes, DNA methylation analysis was performed using the entire pup. We have described it in the Results section (lines 116-118) as follows: Average DNA methylation levels on the entire pup at H19-DMR (m1–m4 sites) were 9–13% in epigenome-edited mice and 43–47% in control mice.*

Comment: Some figures need to be tidied up and neatly aligned. It seems that some legends, scale bars, etc. shifted (perhaps during pdf conversion).

*Response: We thank the reviewer for this comment. This error has been corrected in accordance with the reviewer's comment.*

Comment: Methods 1&2 do not yet seem an efficient way to generate epi-edited mice. How would the authors propose to improve on this considering that the epigenetic modification will not be passed on to the next generation?

*Response: Thank you very much for the helpful suggestion. As you mentioned, the epigenetic modification will not be passed on the next generation in methods 1 & 2. We would like to propose future plans to improve methods 1 & 2. For example in method 1, vector-integrated ES cells can be used for generation of germline chimeras by aggregation with preimplantation embryos. In this case, epigenetic modification will be passed on the next generation. In method 2, degradation of injected RNA occurred during 3 days in embryos; therefore, use of more stable RNA which has nuclease-resistance modification could increase editing efficiency.*

Comment: Although stable integration occurs in the same locus (Rosa26) for all epi-edited mice, there is variation in dCas9 expression. Ca

*Response: We examined whether vector was exactly integrated into the Rosa26 locus or not by PCR analysis, and we found that the vector was not integrated into Rosa26 locus but other loci; therefore, this could cause the variation of dCas9 expression. We have added Supplementary Fig. 5 and the description in Results (lines 191-193) as follows: Unexpectedly, PCR analysis clarified that the vector was not integrated into the Rosa26 locus but other loci in all vector integrated mice (Supplementary Fig. 5), indicating variation of dCas9 expression was caused by vector-integrated loci.*


Reviewer #2: In this manuscript, Horii et al generate a mouse model for an imprinting disorder, Silver-Russel Syndrome, by targeted demethylation of the H19 differentially methylated region (H19-DMR). The authors use a dCas9-SunTag and scFv-GFP-TET1 based system to perform the epigenome editing by 3 different methods using mouse embryonic stem cells or fertilized eggs. In the first method, the authors perform tetraploid complementation that enables generation of mice derived entirely from epigenome edited ES cells. In the second method, the authors overcome the limitation of absence of ESC lines in non-rodent animals by transiently expressing the mRNA for epigenome editing in fertilized eggs to generate epigenome edited mice. A major limitation of both the above methods is that the epigenome editing does not undergo germline transmission. In the third method, the authors overcome this hurdle by stably expressing the epigenome editing factors by targeting them to the Rosa26　locus. Additionally, the authors have demonstrated that their method is also effective in human cells by replicating their experiment in HEK293 cells.

Major points:
Comment: COBRA detects the methylation status at the recognition sites of the restriction enzymes, and therefore is not the best tool to assay for DNA methylation in this study. Because the entire paper is based on the epigenome editing, it is imperative for the authors to validate their experiments using a method that comprehensively determines the DNA methylation across the entirety of H19 promoter and H19 differentially methylated region. Ideally, the authors would use a sequencing-based approach such as amplicon sequencing to validate their demethylation experiments.

*Response: We agree with your suggestion. Therefore, we have comprehensively determined the DNA methylation of 145 CpG sites across 15 kb regions including the entirety of H19-DMR and promoter region by amplicon sequencing analysis for representative sample in all three methods (Fig. 5). We have added following descriptions in Results: “To compare comprehensive DNA methylation status among the three methods, amplicon sequencing analysis was performed for 145 CpG sites across 15 kb regions around H19-DMR (Fig. 5). H19-DMR, promoter and gene body regions were hypomethylated in all epigenome-edited newborn mice as expected. Among these samples, DNA methylation pattern was similar, and did not depend on gRNA (H19-DMR or H19-DMR+promoter) or method used (lines 204-209).”*

Comment: Apart from measuring the body weight, blood glucose level and cardiac fibrosis the authors do not perform a thorough phenotypic analysis of the mice obtained through each of the 3 methods. Because the objective of this paper is to generate a mouse model for an imprinting disorder, the authors should perform a deeper phenotypic analysis to show the amount of phenotypic overlap between their model and patients with SRS. The authors could qualify their claims for the model, and/or determine the Incidence of some of the additional phenotypes seen in patients such as macrocephaly, body asymmetry, feeding difficulties, clinodactyly, learning difficulties, and maxillofacial abnormalities.

*Response: We greatly appreciate the reviewer's comment on this point. In the revised manuscript, we examined some of the additional phenotypes seen in patients such as feeding difficulties (Fig. 6d), maxillofacial abnormalities (Fig. 7a), macrocephaly (Fig. 7b, 7c), body asymmetry (Fig. 7d), and clinodactyly (Supplementary Fig. 8a).*

Comment: In Figs 2b, 3c, 3d, and 5c the methylation level at only one of the 4 CpG sites (m2) has been determined. Is there a reason why only one of the sites was chosen for the methylation analysis? The methylation level of m2 site might not accurately reflect the methylation level across the entire H19-DMR. At the very least, testing the methylation levels in all the 4 sites is necessary for such a claim.

*Response: We thank the reviewer for this comment. We have performed COBRA analysis for m1-m4 and p1-p4 sites in these Figures (Fig.2b, 3c, 3d and 6c (former 5c)).*

Comment: The authors need to demonstrate germline transmission of the H19-DMR demethylation in mice which stably express the epigenome editing factors to validate their claim in discussion.

*Response: We thank the reviewer for this comment. We performed DNA methylation analysis of F1 pups, and found apparent DNA demethylation in vector integrated mice (Supplementary Fig. 9, lines 238-243).*

Minor points:
Comment: In Fig 2d the authors have determined the methylation level of newborn mice at the 4 CpG sites. Have the authors investigated if this trend continues during the postnatal development of the mice?　

*Response: Thank you for the helpful comment. Indeed, it is interesting to examine whether introduced demethylation in H19-DMR continues during the postnatal development or not. However, we did not perform postnatal DNA methylation analysis in this study. We would like to give it more consideration moving forward.*

Comment: The level of overexpression of H19 and downregulation of Igf2 in the unedited mouse appears to be comparable to that of the epigenome edited mice. Could the authors comment on this apparent discrepancy?

*Response: Thank you for the helpful comment. As the reviewer indicated, H19 expression level is comparable between epigenome edited mice and the control mice in method 2 (Fig. 3e) while H19 is significantly upregulated in epigenome edited mice of method 1 (Fig. 2e) and 3 (Fig. 4c). Although the reason for this is not clear, only one mouse showed robust demethylation of H19-DMR in method 2 (Fig. 3d) while at least two mice showed robust demethylation in method 1 (Fig. 2d) and 3 (Fig. 4b). We have added the description in Results (lines 160-164).*

Comment: In Fig3c legend, it is mentioned that the graph represents methylation at m1 and m2 contradicting the text.

*Response: We thank the reviewer for this comment. We corrected the contradiction in the text. In the revised manuscript, we additionally examined the methylation at m1-m4 and p1-p4 and changed the text according to this (line 781).*

Comment: In Fig 3d, how did the authors decide 35% methylation as a cutoff for delineating methylated and demethylated H19-DMR?

*Response: We sincerely apologize for any confusion. We decided cutoff between methylated and demethylated as the lowest methylation level (35%) in control mice at the m2 site in H19-DMR. In the revised manuscript, DNA methylation analysis was carried out for other 6 sites (m1, m3, m4, p1, p2 and p4). As a result, these 4 mice surely showed demethylation in each site compared to control mice (Fig. 3d arrows).*

Comment: In the discussion, vector-integrated mice not showing H19-DMR demethylation having no Cas9 expression is not shown in Supp Fig6e, as is stated, but is shown in Sup Figure 5e.

*Response: We thank the reviewer for this comment. This error has been corrected in accordance with the reviewer's comment (New Supplementary Fig. 4e; line 357-359).*

**Second round of review**

**Reviewer 1**

The authors have addressed all questions and added new data, most convincingly comprehensive DNA methylation was added (Fig 5).

**Reviewer 2**

All of our major and minor comments have been well addressed. We have no further concerns in the text. However, we suggest authors to perform two more minor changes in main figures.

1. The two-head yellow arrows in the micro-CT photo of TG5 mice in Figure 7d are not well marked. The short one did not match the ends of humerus bone. The authors need to re-mark arrow and re-calculate Right/Left Ratio.

2. The body weight change in Figure 2f (Method 1) is displayed as bar chart, while the weight change in Figure 3f and 4d (Method 2 and 3) are dot diagrams. This would be better if the display format was unified.

**Authors Response**

**Point-by-point responses to the reviewers’ comments:**

Reviewer #2: All of our major and minor comments have been well addressed. We have no further concerns in the text. However, we suggest authors to perform two more minor changes in main figures. *Response: We greatly appreciate the reviewer’s insightful comments about our study, which have helped us to significantly improve our paper.*

Comment 1: The two-head yellow arrows in the micro-CT photo of TG5 mice in Figure 7d are not well marked. The short one did not match the ends of humerus bone. The authors need to re-mark arrow and re-calculate Right/Left Ratio.

*Response: We sincerely apologize for any confusion. We have re-marked arrows (Fig. 7d). In contrast, these arrows are just two-dimensional illustrations that approximately show humerus length, but do not show precise length. We measured precise length of humerus by each pair of landmarks using the VGStudio MAX2.2 software.*

Comment 2: The body weight change in Figure 2f (Method 1) is displayed as bar chart, while the weight change in Figure 3f and 4d (Method 2 and 3) are dot diagrams. This would be better if the display format was unified.

*Response: Thank you very much for the helpful comment. We have changed Fig. 2f from bar chart to dot diagram.*
